# Genetic and Environmental Variation in Continuous Phenotypes in the ABCD Study®

**DOI:** 10.1007/s10519-022-10123-w

**Published:** 2022-11-10

**Authors:** Hermine H. M. Maes, Dana M. Lapato, J. Eric Schmitt, Monica Luciana, Marie T. Banich, James M. Bjork, John K. Hewitt, Pamela A. Madden, Andrew C. Heath, Deanna M. Barch, Wes K. Thompson, William G. Iacono, Michael C. Neale

**Affiliations:** 1 Department of Human and Molecular Genetics, Virginia Institute for Psychiatric and Behavioral Genetics, Virginia Commonwealth University, PO Box 980033, Richmond, VA 23298-0033 USA; 2 Department of Psychiatry, Virginia Institute for Psychiatric and Behavioral Genetics, Virginia Commonwealth University, Richmond, VA USA; 3 Massey Cancer Center, Virginia Commonwealth University, Richmond, VA USA; 4 Departments of Radiology and Psychiatry, University of Pennsylvania, Philadelphia, PA USA; 5 Department of Psychology, University of Minnesota, Minneapolis, USA; 6 Department of Psychology and Neuroscience, University of Colorado, Boulder, USA; 7 Institute of Cognitive Science, University of Colorado, Boulder, USA; 8 Institute for Behavioral Genetics, University of Colorado, Boulder, USA; 9 Department of Psychiatry, Washington University in St Louis, St Louis, MO USA; 10 Division of Biostatistics and Department of Radiology, Population Neuroscience and Genetics Lab, University of California at San Diego, La Jolla, CA USA

**Keywords:** ABCD, Adolescence, Twin, Heritability, Environment, Neuroscience, Genetics, Cognition, Cognitive abilities, Personality, Psychiatric disorders, Substance use, Children, Open science, FAIR data

## Abstract

Twin studies yield valuable insights into the sources of variation, covariation and causation in human traits. The ABCD Study® (abcdstudy.org) was designed to take advantage of four universities known for their twin research, neuroimaging, population-based sampling, and expertise in genetic epidemiology so that representative twin studies could be performed. In this paper we use the twin data to: (i) provide initial estimates of heritability for the wide range of phenotypes assessed in the ABCD Study using a consistent direct variance estimation approach, assuring that both data and methodology are sound; and (ii) provide an online resource for researchers that can serve as a reference point for future behavior genetic studies of this publicly available dataset. Data were analyzed from 772 pairs of twins aged 9–10 years at study inception, with zygosity determined using genotypic data, recruited and assessed at four twin hub sites. The online tool provides twin correlations and both standardized and unstandardized estimates of additive genetic, and environmental variation for 14,500 continuously distributed phenotypic features, including: structural and functional neuroimaging, neurocognition, personality, psychopathology, substance use propensity, physical, and environmental trait variables. The estimates were obtained using an unconstrained variance approach, so they can be incorporated directly into meta-analyses without upwardly biasing aggregate estimates. The results indicated broad consistency with prior literature where available and provided novel estimates for phenotypes without prior twin studies or those assessed at different ages. Effects of site, self-identified race/ethnicity, age and sex were statistically controlled. Results from genetic modeling of all 53,172 continuous variables, including 38,672 functional MRI variables, will be accessible via the user-friendly open-access web interface we have established, and will be updated as new data are released from the ABCD Study. This paper provides an overview of the initial results from the twin study embedded within the ABCD Study, an introduction to the primary research domains in the ABCD study and twin methodology, and an evaluation of the initial findings with a focus on data quality and suitability for future behavior genetic studies using the ABCD dataset. The broad introductory material is provided in recognition of the multidisciplinary appeal of the ABCD Study. While this paper focuses on univariate analyses, we emphasize the opportunities for multivariate, developmental and causal analyses, as well as those evaluating heterogeneity by key moderators such as sex, demographic factors and genetic background.

**Supplementary Information:**

The online version contains supplementary material available at 10.1007/s10519-022-10123-w.

## Introduction

Efforts to understand the origins of human individual differences have included an enormous variety of physical, behavioral and psychological traits. Part of this effort has focused on distinguishing between genetic and environmental sources of variation and *quantifying* the relative contributions of genetic and environmental factors, which may be measured directly or inferred from the resemblance between relatives. This approach has been fruitful because individual differences in almost all traits are likely caused by both genetic and environmental factors. While twin, adoption and extended pedigree data may be used to disaggregate and estimate the relative contributions of genetic and environmental factors to trait variance, the classical twin study (CTS) has proved to be the dominant design. The rationale underlying the CTS is that the difference in phenotypic similarity between monozygotic (MZ; identical) and dizygotic (DZ; fraternal) twin pairs can be used to infer and distinguish the influences of environmental and genetic factors. An advantage of using both MZ and DZ twin pairs is that the effects of rearing by the same parents in the same household (known as shared or common environment, or ‘C’) may be separately quantified from the effects of environmental events that are unique to each twin (known as specific, unique or individual environment, or ‘E’). These components, along with additive genetic variance (A), referring to the cumulative effects of individual genetic loci, generate the ‘ACE’ model acronym. Prior estimates of additive genetic, common and specific environmental effects based on published studies were comprehensively reported (Polderman et al. 2015), featuring thousands of human traits varying from simple questionnaire items to costly neuroimaging and other in-person assessments. Here, we contribute to the literature by analyzing publicly available data from the first wave of in-person assessments of the Adolescent Brain Cognitive Development Study (ABCD Study®; abcdstudy.org) (Iacono et al. 2018).

The ABCD Study is the largest longitudinal study of child health and development in the United States. The study population comprises 11,880 youth who were 9–10 years old in September 2017 and living in the United States. The cohort was designed to approximate the demographics of the US population, except that twins were oversampled to generate greater statistical power to test hypotheses concerning sources of variation and direction of causation among variables. The ABCD Study offers a heretofore unexplored examination of sources of cognitive and brain feature variability in children as young as 9. A key initial motivation for the project was to quantify the effects of substance use on the brain, cognition, behavior and psychiatric outcomes, so these domains have been carefully assessed. A unique aspect of ABCD is its longitudinal assessment of structural and functional neuroimaging, beginning in pre- or early-adolescence. From its inception, transparency, inclusivity, and accessibility have been emphasized to promote public trust in the ABCD Study. A key component of transparency and accessibility has been an embrace of open science methods, thorough study documentation, and standardized data processing. Open-source software scripts and standardized data processing and analysis pipelines have been made publicly available to facilitate data use and access by researchers unaffiliated with ABCD. All the neuroimaging data, genotypic measures, and questionnaire responses have been processed centrally rather than by individual research teams or study sites. Such standardization is a critical part of ensuring robust quality control. To date, no comparable standardized processing has been performed for the data from the twin study embedded within ABCD.

This paper and its companion supplementary documents detail the comprehensive and systematic data processing and analysis performed for the 53,000 variables collected during the first study wave in order to create and introduce here to the community a novel online web tool that will enable scholars from many backgrounds (not just genetics) who are interested in the relative degrees to which individual variation in children’s mental, physical, or brain structural traits is likely caused by genetic or environmental factors to peruse the results for selected phenotypes on demand. Given the broad scope of the ABCD Study assessments, a thorough review of relevant literature on sources of variability (e.g., heritability) for all domains of measurement would be prohibitively long for publication in a single research article. Instead, interested readers can refer to concise reviews of prior genetically-informative studies of each primary domain, including structural neuroimaging, in our online supplementary material.

We begin here by summarizing basic twin methodology and describing the ABCD twin dataset. We then explain our analysis methods in detail, followed by a results section composed of commentary on key results from the twin modeling, along with a link to an omnibus online table of results. We conclude with commentary on the assumptions of the classical twin study as they relate to ABCD data, as well as a brief discussion of similarities and differences between the ABCD results and the extant literature for four key domains—brain structural, cognitive, psychiatric, and morphometric features (functional imaging will be addressed in a future article)—and offer suggestions for future avenues of exploration. Specifically, the discussion will review previous findings on the components of variance of four phenotypic domains: structural neuroimaging, neurocognition, child psychopathology, physical and other traits captured in ABCD, where childhood findings effectively formed our expectations as we applied ACE modeling to the ABCD twin dataset. We then review how the results from analyzing the ABCD data using a consistent variance components approach compare with previous findings, highlight some novel findings and thus provide a foundation for multivariate, developmental, causal and moderation applications evaluating the role of gender, race/ancestry and socioeconomic status (SES). For additional information about the ABCD Study, a key literature resource for the rationale, instrument selection, data processing and interpretation of ABCD Study results is the 2018 special issue (Vol. 32, pp. 1–164) of Developmental Cognitive Neuroscience (Feldstein Ewing et al. 2018). Particularly relevant to the present article is the Iacono et al. 2018 paper discussing the special value of twins in the ABCD Study.

## Methods

### Twin recruitment

The ABCD Study® operates as a consortium, comprising 21 data collection sites across the continental US to sample in an epidemiologically informed and inclusive way. By design, the ABCD Study® over-sampled twin pairs at four sites known for their curation of population-based twin samples: the University of Minnesota, Washington University St. Louis, the University of Colorado Boulder, and Virginia Commonwealth University. All four twin sites ascertained twins from state birth records (Iacono et al. 2018). Although twins were recruited opportunistically at the other 17 ABCD sites, their numbers are relatively modest. That data is not included in the current analyses. This strategy restricts our twin samples to those ascertained in a systematic way from their birth cohorts in each of the four twin site states (Minnesota, Missouri, Colorado, and Virginia). Also, by design, only same-sex twin pairs were ascertained, as it was deemed not feasible to obtain enough opposite-sex twin pairs to have sufficient power to detect qualitative sex differences in sources of variation.

### Participants

Participants for this study include all same-sex twin pairs (N = 772) from the four ABCD ‘twin sites’, namely the University of Colorado Boulder (CUB), the University of Minnesota (UMN), Virginia Commonwealth University (VCU) and Washington University at St. Louis (WUSTL), for whom zygosity was assigned using genomic data from the smokescreen array (Baurley et al. 2016). Sample sizes by site, zygosity and sex are in Table 1 and by race/ethnicity in Table 2. Data were included from the baseline assessment of 1,544 twins at 9–10 years of age. The current twin sample was less racially diverse; their parents were slightly more educated, more likely to be married and had higher incomes than the full ABCD sample (see Supplemental Table 1, and (Iacono et al. 2018) for details on ascertainment and power). All procedures were approved by a central Institutional Review Board (IRB) at the University of California, San Diego, and in some cases by individual site IRBs (e.g. WUSTL) (Auchter et al. 2018). Parents or guardians provided written informed consent after the procedures had been fully explained; children assented before participation in the study (Clark et al. 2018).

**Table 1:** Number of twin pairs by site, zygosity and sex in the ABCD study

	CUB	UMN	VCU	WUSTL	Total
MZm	49	49	47	28	173
DZm	41	44	46	32	163
MZf	54	51	46	67	218
DZf	53	45	55	65	218
Total	197 (26%)	189 (24%)	194 (25%)	192 (25%)	772
DZos	0	1	1	0	
na	19	27	21	25	92

Only the four primary twin sites are shown: University of Colorado Boulder; University of Minnesota; Virginia Commonwealth University; and Washington University, St. Louis, denoted CUB, UMN, VCU and WUSTL, respectively

*MZ* monozygotic, *DZ* dizygotic, *m* male, *f* female, *os* opposite sex, *na* zygosity not available

**Table 2:** Number of individual twins with known zygosity by site and race/ethnicity in the ABCD study

	CUB	UMN	VCU	WUSTL	Total
White	252	296	216	259	1023 (66%)
Black	8	30	100	82	220 (14%)
Hispanic	86	23	28	15	152 (10%)
Asian	0	1	2	0	3 (0%)
Other	48	28	42	28	146 (9%)

### Measures

In keeping with NIH’s policy of zero embargo time for investigators collecting the data, only publicly available data from the NIMH National Data Archive (NDA) are analyzed, specifically the baseline wave of data collection from the ABCD 3.0 data release. All continuous variables, and those with more than twelve categories were included in the analyses, totaling 53,172 variables. We focus the discussion of the results on 14,500 variables, covering structural neuroimaging, neurocognition, childhood psychopathology, physical and other traits. Note that below we only describe the ABCD capture of the most commonly-used phenotypes. Conversely, tables in our Supplemental Online Resource (https://abcdtwinhub.shinyapps.io/baselineTwinResults) includes results from *all* tabulated (i.e., pre-calculated) continuously distributed variables in the open access 3.0 dataset, described in the ABCD Data Release documentation, including those discussed here, and an additional 38,672 task Magnetic Resonance Imaging (MRI) related variables. The online resource includes an app that allows the user to search for specific variables and select specific output columns for display. A second app generates brain images with selected parameters. Additional figures graphing ACE results are also included.

#### ABCD baseline structural neuroimaging battery

Extensive details of the ABCD neuroimaging acquisition sequences and processing streams have been presented elsewhere (Casey et al. 2018; Hagler et al. 2019). Our analyses focused on metrics extracted from the Desikan-Killiany (DK) and Destrieux Atlases implemented in Freesurfer, with evidence that identification of the features on which these atlases are based works well down to age 4 (Ghosh et al. 2010). The ABCD imaging protocol was harmonized across data collection sites for three 3T scanning systems (Siemens Prisma, Philips, General Electric 750), all of which used standard adult-size multi-channel head coils and multiband echo planar imaging (EPI) acquisitions. The diffusion MRI acquisition had high resolution (1.7 mm isotropic voxels) and utilized multiband EPI (Hagler et al. 2019). The scanning sequences that yield structural data (Casey et al. 2018) include a localizer, T-1 weighted scan, diffusion tensor imaging (DTI), and T-2 weighted scans. Real-time motion detection and correction during acquisition are implemented by customized hardware and software. Imaging parameters were harmonized as much as possible between scanner manufacturers.

#### ABCD baseline neurocognition battery

The NIH Toolbox®–Cognition battery (NIHTB-CB, herein referred to as “the Toolbox”) was administered and includes seven tasks that measure episodic memory (NIHTB-CB Picture Sequence Memory), executive function (NIHTB-CB Dimensional Change Card Sort), attention (NIHTB-CB Flanker Task), working memory (NIHTB-CB List Sorting Working Memory), processing speed (NIHTB-CB Pattern Comparison Processing Speed), and language abilities (NIHTB-CB Picture Vocabulary & Oral Reading Recognition). We report here on the uncorrected Toolbox scores. Composite indices of fluid and crystallized reasoning are also derived (Bleck et al. 2013; Gershon et al. 2013a, b; Hodes et al. 2013). The neurocognitive battery has been more extensively described elsewhere (Luciana et al. 2018; Thompson et al. 2019). The Supplemental Materials section provides brief descriptions of each task.

Because the Toolbox measures do not employ delayed recall or recognition trials as part of their memory assessments, a customized automated version of the Rey Auditory Verbal Learning Test (RAVLT), a widely used test of auditory learning and memory, was implemented for this purpose. An automated version of the Matrix Reasoning subtest from the Wechsler Intelligence Test for Children-V (WISC-V)(Wechsler 2014) was administered in standardized format using automated technology [Q-interactive (Daniel et al. 2014)]. The Little Man Task (LMT) (Acker & Acker 1982) was used to assess visual-spatial processing, specifically mental rotation with varying degrees of difficulty. Members of our group have published a principal components analysis of the battery (Thompson et al. 2019). Three principal components were derived from performance on the NIH Toolbox, RAVLT, and LMT measures: they reflect General Ability, Executive Function, and Learning/Memory, respectively.

#### ABCD childhood psychopathology battery

In the baseline assessment of 9- and 10-year-old children, parents rate their children’s behavior using the Child Behavior Checklist [CBCL: (Achenbach & Edelbrock 1981, 1983)]. The 112-item checklist yields an overall Externalizing and Internalizing behavior score, as well as subscale scores. Subscale scores include DSM5-oriented Attention-Deficit Hyperactivity Disorder (ADHD), Anxiety Disorder, Conduct Disorder, Depression, Oppositional behavior, and Somatic Problems. Subscale syndrome scores include those for aggressive behavior, anxiety/depression, attention, rule breaking, social difficulties, somatic problems, thought problems, and withdrawn depression, as well as other problems and an overall total problem score. Additional scale scores are available for Sluggish Cognitive Tempo, Obsessive–Compulsive Problems, and Stress. Each scale score is available as a raw score summation and as a transformed t-score used more commonly by clinicians to provide interpretive guidance. We would expect raw scores and t-scores to behave similarly in most applications or analyses and report results here for raw scores. For the teacher ratings [assessed with the Brief Problem Monitor (BPM): (Achenbach et al. 2011)], the 18-item checklist was scored for the overall internalizing and externalizing scales, attention problems, and a total problem score.

#### ABCD physical and other traits

We report here on physical and other traits that are measured as continuous variables in ABCD. Standardized measurements following CDC guidelines were used to monitor health, obesity, growth and physical development [CDC (Division of Nutrition) 2016], including height, weight, body mass index, and waist circumference. Pubertal hormones were assessed through the collection of a single salivary biospecimen from which salimetric scores for DHEA, testosterone and estradiol were derived. The Sleep Disturbance Scale for Children (SDSC) (Bruni et al. 1996) comprehensively screens for a variety of sleep disturbance types using a 26-item Likert-type rating scale administered to a parent. Six scales (disorders of initiating and maintaining sleep, sleep breathing, arousal or nightmares, sleep wake transition, excessive somnolence, and sleep hyperhidrosis) and an overall sleep–wake disturbance symptom severity score were derived. A measure of visual media use (Sharif et al. 2010) included questions on the overall amount of time that the youth spends using visual media during typical weekdays and weekend days. Extracurricular activities, including sports, music related activities, or hobbies, were assessed with the parent-reported Sports and Activities Involvement Questionnaire (Huppertz et al. 2016). In this report, we included continuous measures of years and hours per week of music listening and reading for pleasure. See (Barch et al. 2018) for more details on measures.

### Statistical methods

Prior to twin modeling, the effects of sex at birth, age, race/ethnicity (using four dummy variables comparing Black, Hispanic, Asian, and Other to the white reference group [the largest group]), and site (using three dummy variables comparing CUB, VCU and WUSTL to the UMN reference group) were regressed out for each variable. Residuals were then standardized and outliers (values greater than four standard deviations from the mean) set to missing prior to biometric analyses. Pearson product-moment correlations were calculated separately by zygosity and sex for the standardized residuals of each of the measures, adjusted for sex, age, race/ethnicity and site. Homogeneity of variances by zygosity and sex was tested with the Levene test (Soave & Sun 2017). Standard biometrical multi-group saturated and ACE models (Neale & Cardon 1992) were fitted to all measures, directly estimating ACE variance components, and likelihood-based confidence intervals for standardized variance components. For each variable, we fitted five models: 2-group saturated and ACE models with male and female twins combined in MZ and DZ groups, and 4-group saturated and two ACE models, one allowing ACE components to differ by sex and another constraining them to be the same across sex to test the significance of heterogeneity by sex. The comparison of the saturated with the ACE models assesses whether the assumptions of equal means and variances across twin order and zygosity are met. All analyses were performed in R version 4.1.1 (R Core Team 2017) and using OpenMx version 2.20.09 (Boker et al. 2011; Neale et al. 2016). In this article we discuss only results of variance component analyses with male and female pairs combined, although the Supplemental Online Resource contains these more extensive analyses.

#### Variance components model

We chose to follow Verhulst & Neale’s (Verhulst et al. 2019) recommendation to estimate variance components instead of path coefficients as parameters for these analyses. This choice, like most, has both advantages and disadvantages. The key difference between the approaches is that direct estimates of variance components may be negative, whereas estimates of path coefficients are squared and therefore necessarily non-negative when generating the expected covariance matrix. Thus, the path coefficient approach has an implicit lower bound of zero for variance associated with A, C or E. Since variance components are themselves squared quantities, this restriction seems rational, and has been applied for most behavior genetic analyses over the past 25 years. However, there are disadvantages to bounding the estimates, particularly for hypothesis testing and meta-analyses. First, consider the distribution of estimates of A when the null hypothesis that A = 0 is true. MZ and DZ correlations are predicted to be equal. Random sampling variation would cause the rMZ and rDZ to vary around the same value, with the expectation that 50% would find rMZ > rDZ and 50% where rMZ < rDZ. The former case would yield a positive estimate of A, and the latter a negative one—unless the path coefficient approach is used, in which case it would hit the effective lower bound of zero. Direct variance component estimates across multiple studies would correctly average estimates of A to be zero. The path coefficient approach, however, would yield an average A that is greater than zero. The less precise the estimates are, the greater their variance, so the greater the upward bias. This trend occurs because the proportion of estimates that fall below zero (the lower tail of the estimates’ distribution) increases with the variance when the mean estimate is greater than zero. Before “heritability deniers” complain of systematic upward bias to A from prior twin studies, it should be noted that estimates of C are subject to the exact same type of bias.

While the classical twin design can estimate two more components of variance (A and C) than a study of unrelated (and not genotyped) individuals (which estimates only E), it cannot simultaneously estimate a fourth, D, representing dominance genetic effects. These effects are expected to correlate perfectly between MZ twins, and 0.25 between DZ twins, but they are confounded with C. Choosing between ACE and ADE models might be done on the basis of prior studies, or by inspecting the twin correlations in the current one. Selecting either C or D obscures the important point that both variance components may simultaneously contribute to variation. Historically, the practice was to inspect the MZ and DZ correlations and to fit an ADE model if rDZ < 0.5rMZ, but to fit an ACE model otherwise. That procedure results in models that are more likely to fit the data and to produce non-zero estimates, but it unfairly capitalizes on the prior knowledge of the correlations. Here we chose to fit ACE models by direct variance estimation for all variables, regardless of the pattern of twin correlations, as it allows us to calculate the variance components we would have obtained when fitting an ADE model. The variance components approach is, from a statistical standpoint, much superior to the path coefficients approach, because its Type I and II error rates are correct. The apparent disadvantage, however, is that negative variance component estimates are both possible and more difficult to interpret. We therefore suggest that the C component be interpreted as a residual component of familial variance that is the sum of the effects of shared environment and non-additive genetic variance. Negative C suggests that non-additive genetic factors have outweighed any effects of the family environment. Positive C indicates the opposite. If the likelihood-based confidence intervals of C overlap zero, it may be because neither C nor D influence the phenotype, or because the two counterbalance each other to yield a non-significant estimate. This apparent difficulty in interpretation should be recognized as an opportunity to adopt a broader perspective on the nature of variance component estimates from twin studies.

#### Interpreting classical twin study results

We now illustrate the interpretation of the results with two examples of applying the variance components modeling approach to the anthropometric data (see also Fig. 8). For weight, the MZ correlation (rMZ = 0.89 and the DZ correlation (rDZ) = 0.46. This pattern of twin correlations is consistent with a model containing A, C and E sources of variance, i.e., an ACE model, as rDZ is slightly greater than half rMZ. The maximum likelihood estimates of the proportions of variance accounted for by A, C and E are respectively 0.88, 0.01 and 0.11, close to what one would have obtained by applying Falconer’s (Falconer & Mackay 1998) estimate of heritability as 2(rMZ-rDZ). For waist circumference, rMZ = 0.73 and rDZ = 0.34, which are not consistent with an ACE model but rather an ADE model, as rDZ is less than half rMZ. This pattern of correlations is reflected in a negative estimate for C (0.-09) and a larger estimate of A (0.83) with the E estimate is still close to 1 minus rMZ (0.26). However, we can calculate the corresponding estimates of A, D and E from the estimates of the ACE model as done here. The proportion of variance associated with A, under an ADE model, can be calculated as A + 3C divided by the total variance A + C + E; and, similarly, the proportion of variance due to D is calculated as -2C/(A + C + E). For waist circumference, the estimate of A would thus be 0.57 and the estimate of D 0.17 with E remaining at 0.26, which are more readily interpretable. In a similar way, one could recalculate values for A, C, and E from ADE estimates if an ADE model had been fitted to the data instead, with C = -0.5D and A’ = A + 1.5D. Ideally, data from other types of pairs of relatives should be collected to resolve these alternatives.

It is important to note that heritability estimates from both twin and genome-wide association studies are population- and measurement-specific. This quality makes heritability estimates for multiple, apparently related traits measured in the same cohort especially informative, as they are derived from the same point in time, and with the same population. Cai et al. illustrated this phenomenon in a 2020 publication comparing heritability estimates for five different measures of major depression in the UK Biobank (Cai et al. 2020). Their team observed substantially different heritability estimates for clinical depression and self-reported depression (~ 31% and ~ 10%, respectively). These results indicate that these phenotypic definitions of depression are not interchangeable for genetic studies, which is a valuable insight for designing future studies. Also important is that the parameter estimates from twin studies should not be interpreted as concrete or absolute. For example, if five twin studies of height are conducted in regions that vastly differ in their access to food and health care, one should expect to observe differences in heritability estimates; however, those differences do not mean that the genetics of height vary by population. Heritability is a proportion. Differences in heritability estimates across populations indicate that the proportion of trait variance attributable to genetic factors varies with respect to the magnitude of influence from environmental factors.

## Results

### Structural neuroimaging

#### Regional measures of morphometry (Figs. 1, 2, 3)

**Fig. 1:**
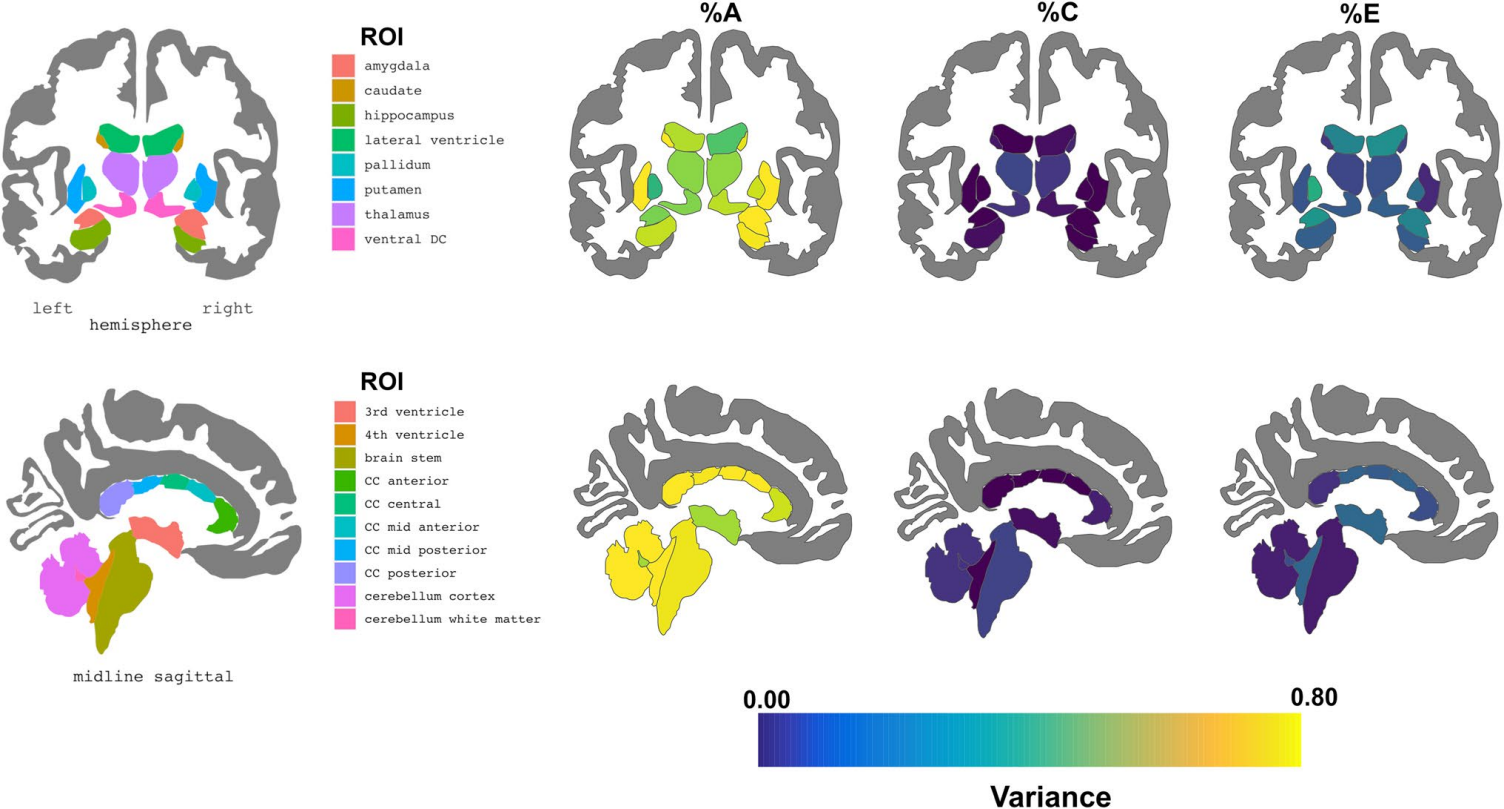
Genetic influences on subcortical, callosal, ventricular, and posterior fossa volumes

As shown in heritability estimates tabulated in Supplemental Online Resource at https://abcdtwinhub.shinyapps.io/baselineTwinResults, ABCD data were consistent with prior findings of high heritability of overall measures of brain morphometry, with rMZ being about twice rDZ, and genetic variance accounting for close to 90% of the variance for overall brain volume (*h*^*2*^ = 94%) and surface area (*h*^*2*^ = 95%). There was lower genetic influence on cortical thickness (*h*^*2*^ = 66%), consistent with prior reports of developmental effects and lower *h*^*2*^ in childhood than adulthood (Rimol et al. 2010; Schmitt et al. 2014; Teeuw et al. 2019). Of interest is whether these genetic effects on cortical thickness differ for posterior brain regions that develop earlier as compared to later developing frontal regions. In this sample there was no evidence to suggest such a difference (left frontal *h*^*2*^ = 58%, right frontal *h*^*2*^ = 61%; left occipital *h*^*2*^ = 58%, right occipital *h*^*2*^ = 54%; left parietal *h*^*2*^ = 56%, right parietal *h*^*2*^ = 55%).

Variance components for selected subcortical, ventricular, corpus callosum (CC), and posterior fossa volumes are summarized in Fig. 1. Similar to prior studies (Blokland et al. 2012; Peper et al. 2007; Schmitt et al. 2007a, b), volumes of the deep gray nuclei were, in general, highly heritable. Approximately 65% of the phenotypic variation in thalamic volumes could be attributed to genetic effects, as compared to estimates of 52% in an adult meta-analysis (Blokland et al. 2012) and 64% in the NIH pediatric sample (Schmitt et al. 2007a, b). Heritability of the corpus striatum (caudate *h*^*2*^ > 70%, putamen *h*^*2*^ = 80%) and hippocampi (*h*^*2*^ =  ~ 75%) was even higher. In comparison, the NIH sample reported a heritability of basal ganglia of 69%, and hippocampal heritability has been reported as high as 80% (Patel et al. 2017). The CC, brainstem, and cerebellum also all were highly heritable, with over 70% of the variance attributable to genetic factors. In general, the ventricles had more modest heritability estimates.

**Fig. 2:**
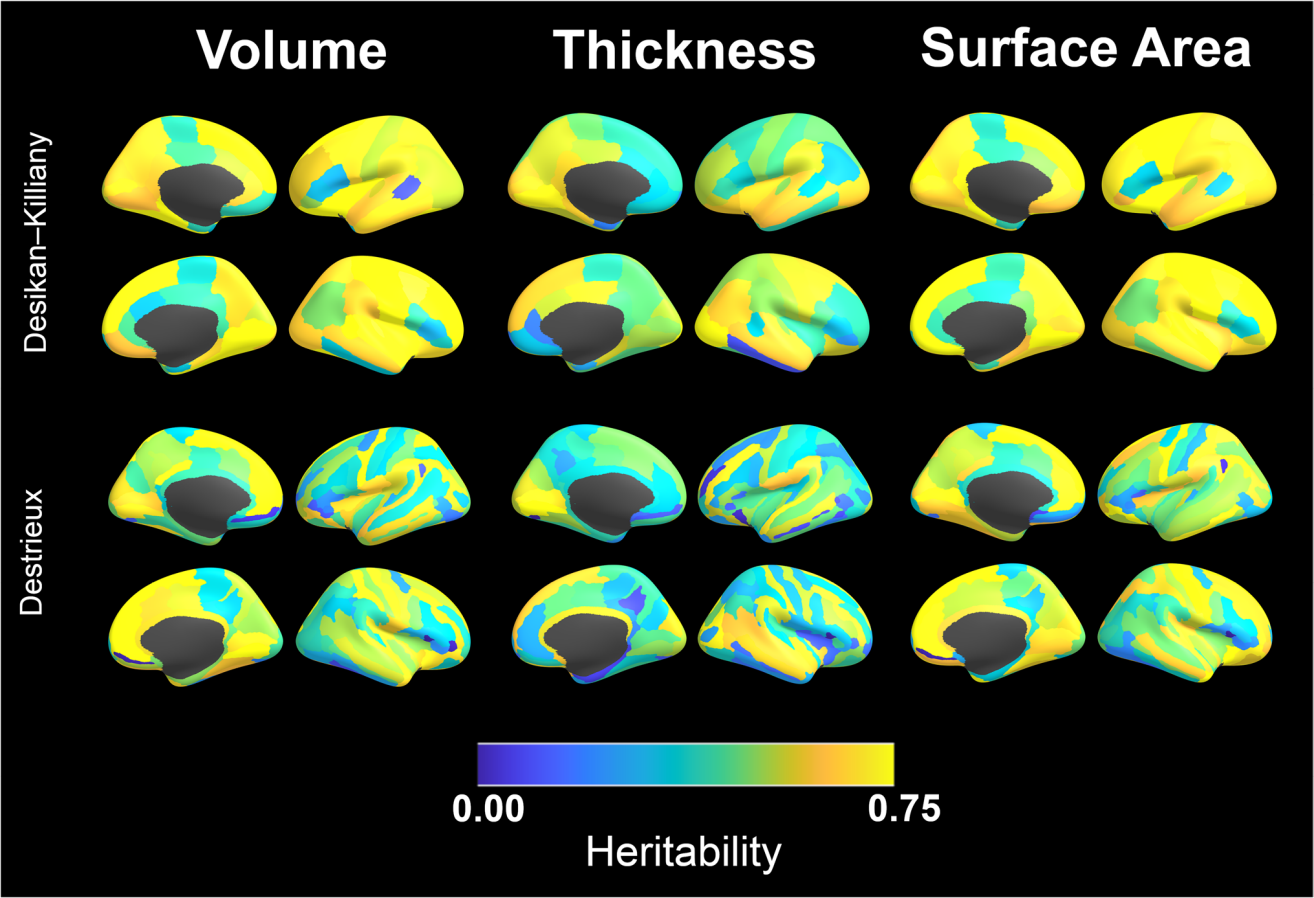
Regional heritability maps for cortical volume, thickness, and surface area. Results using the Desikan-Killiany and Destrieux anatomic parcellations are both provided

Regional heritability maps for the most commonly measured cerebral surface metrics (cortical volume, thickness, and surface area) are provided in Fig. 2. Heritability estimates for cortical (parcel) volumes tended to be greater in the right hemisphere for both atlases. As for global measures, regional measures of cortical thickness were lower relative to other structural endophenotypes, and lower relative to prior adult studies of thickness for both atlases. The higher-resolution Destrieux atlas found that thickness was most heritable in the left supramarginal gyrus, right superior frontal gyrus, right temporal, peri-calcarine, and peri-Rolandic cortex. In general, heritability estimates for thickness were higher in the right hemisphere relative to left. Estimates were also higher in gyri relative to sulci, giving the brain a tigroid appearance in the Destrieux parcellation; mean ROI differences in gyral-sulcal heritability were statistically significant (${F}_{145}^{1}$= 16.1, p-value < 0.0001). Maps of cerebral surface area showed very high heritability estimates for both atlases, In particular, the Destrieux atlas found strong heritability in peri-calcarine cortex extending along the ‘what’ and ‘where’ pathways of the precuneus and occipitotemporal cortex, as well as in the peri-sagittal frontal lobes. Similar patterns were observed for cortical volumes.

**Fig. 3:**
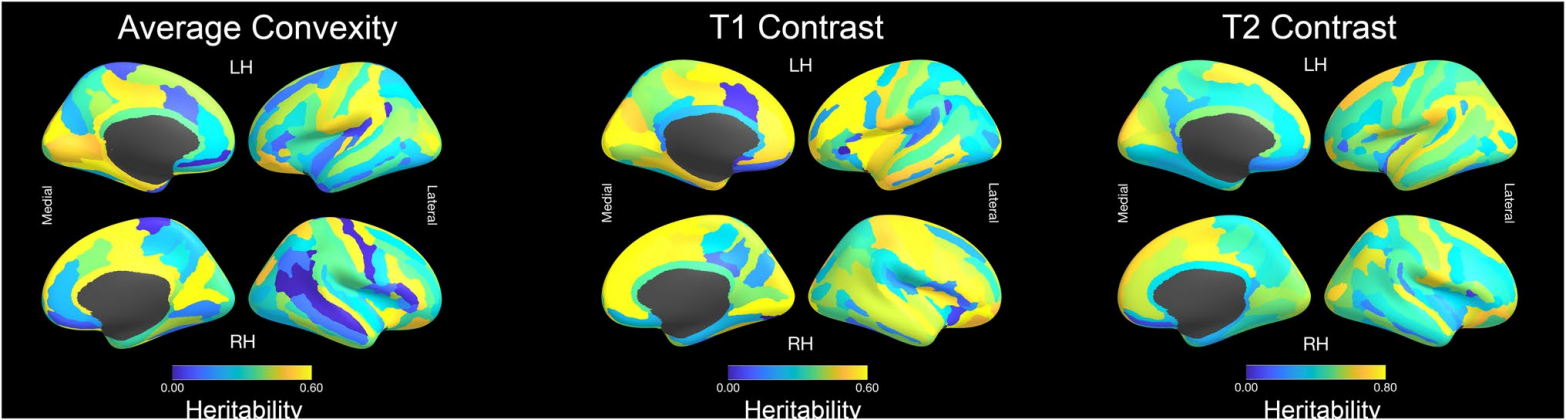
Regional heritability maps for average convexity (a measure of cortical folding), and T1 and T2 contrast (both related to cortical myelination) using the Destrieux parcellation

Results from less commonly investigated structural measures (average convexity, T1 contrast, T2 contrast) are provided in Fig. 3. The heritability of average convexity (a measure of cortical folding) was relatively modest, with *h*^*2*^ estimates for the majority of the cerebrum < 0.40. The principal exceptions were near the central sulcus, calcarine sulcus, and cingulate. T1 and T2 contrast, both potential proxies for cortical myelination, showed high heritability in peri-Rolandic cortex, superior frontal gyri, and peri-cingulate cortex. The T2 contrast heritability estimate was substantially higher than T1’s. There were some similar patterns between T1 and T2 contrast, although with disproportionately higher heritability estimates in the lateral parietal lobes based on T2 image data.

### Anatomical measures of connectivity (Figs. 4 and 5)

**Fig. 4:**
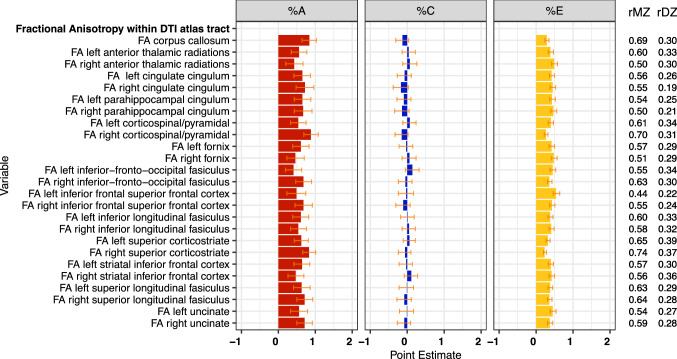
Estimates of genetic (%A), shared (%C) and specific (%E) environmental variance components and MZ and DZ twin correlations for fractional anisotropy

**Fig. 5:**
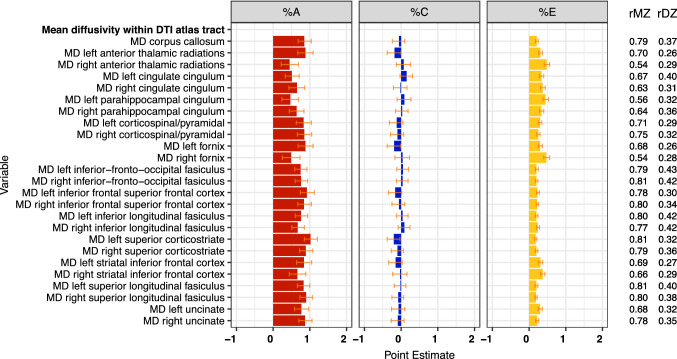
Estimates of genetic (%A), shared (%C) and specific (%E) environmental variance components and MZ and DZ twin correlations for mean diffusivity

For FA values within major white matter tracts, the average estimate of *h*^2^ ranged from 40 to 88% (see Fig. 4) with an average of 62%. For most tracts, rMZs were roughly double rDZs, although dominant genetic effects were suggested for regions such as CC and cingulate gyrus. Heritabilities were highest for regions such as CC, corticospinal tract, superior corticostriate, superior longitudinal fasciculus and uncinate fasiculus. For these latter three pathways, heritabilities were higher for right versus left hemisphere tracts. Heritabilities were lower for anterior thalamic radiation, fornix, inferior longitudinal fasciculus, and inferior frontal-occipital fasciculus. The average heritability for MD across the tracts listed in Fig. 5 was 73%, and values were particularly high for CC and adjacent tracts as well as the superior corticostriate tract, inferior longitudinal fasciculus, superior longitudinal fasciculus, and uncinate fasciculus. Although there were some hemispheric variations in heritability magnitudes, these were not as marked as for FA. For both FA and MD, variations due to shared environmental influences were small while those attributable to unshared environmental factors were moderate ranging from 23 to 56% for FA and from 18 to 49% for MD.

#### Neurocognition (Fig. 6)

**Fig. 6:**
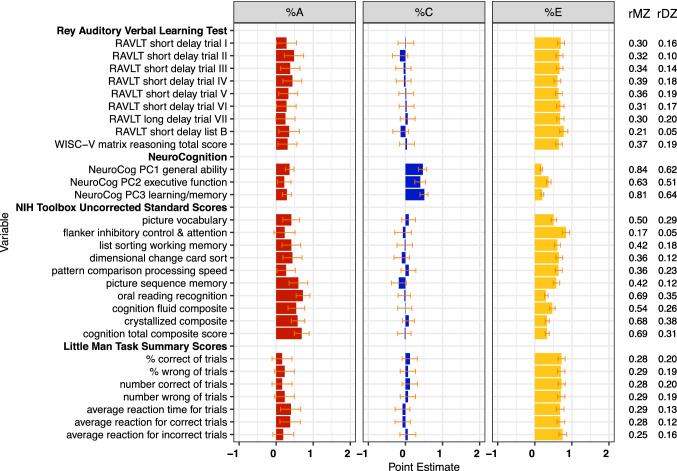
Estimates of genetic (%A), shared (%C) and specific (%E) environmental variance components and MZ and DZ twin correlations for neurocognitive measures

Figure 6 presents findings for key neurocognitive variables, highlighting the primary performance metrics from each task. rMZs (0.17–0.84) were generally higher than rDZs (0.03–0.64), consistent with genetic influence on cognitive performance. Highest rMZs were for the NIH Toolbox crystallized (0.68) and fluid composite (0.54), and derived principal components of general ability (0.85), learning/ memory (0.82), and EF (0.63). However, for the principal components, rDZs are greater than half rMZs, suggesting an impact of shared environment. Notably, rMZs were rather low for some tasks (e.g., Flanker Task; LMT reaction times; Matrix Reasoning Scaled Scores), suggesting substantial influences of unshared environment or measurement error on aspects of EF and visuo-spatial reasoning. There was also some evidence for non-additive genetic influences for the Flanker Task, Dimensional Change Card Sort, Picture Sequencing and performance on several learning trials of the RAVLT.

ACE modeling estimates showed moderate genetic influences on crystallized ability (*h*^2^ = 58%), aggregated fluid abilities (*h*^*2*^ = 57%), and principal-components-based general ability (*h*^*2*^ = 36%). Genetic influences appeared less salient for EF (*h*^*2*^ = 22%) and learning/memory (*h*^*2*^ = 29%). Shared environment estimates were substantial for the three principal components (*c*^*2*^ = 40–50%) but close to zero for the NIH Toolbox uncorrected crystallized and fluid reasoning composites. Non-shared environmental influences were substantial for all composite measures (*e*^*2*^ = 18–47%).

When specific abilities were modeled, heritability estimates were highest for NIH Toolbox Oral Reading (*h*^*2*^ = 74%), Picture Vocabulary (*h*^*2*^ = 40%), Dimensional Change Card Sort (*h*^*2*^ = 44%), and Picture Sequencing (*h*^*2*^ = 61%) tasks. Estimates were low for measures of spatial reasoning (e.g., Matrix Reasoning, Little Man Task: *h*^*2*^ = 16–40%), inhibitory control (Flanker Task: *h*^*2*^ = 24%), processing speed (Pattern Comparison Processing Speed: *h*^*2*^ = 29%) and performance on discrete trials of the RAVLT. The cash choice task (Sparks et al. 2014) was not analyzed here as the measures are ordinal rather than continuous. Shared environmental influences were generally negligible. Consistent with other reports, unshared environmental influences were substantial and, in many cases, relatively large compared to other sources of variation (*e*^*2*^ = 31–81%).

#### Child Psychopathology (Fig. 7)

**Fig. 7:**
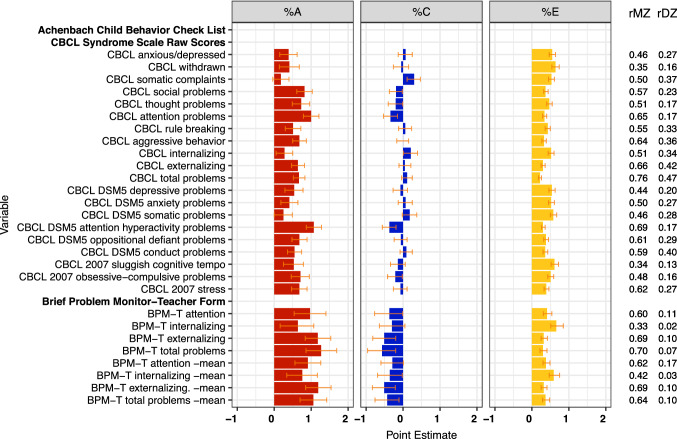
Estimates of genetic (%A), shared (%C) and specific (%E) environmental variance components and MZ and DZ twin correlations for child psychiatric assessments

Results for child psychiatric assessments are presented in **Fig. ****7**. For the CBCL Total, Externalizing, and Internalizing scale scores, the overall MZ correlations for these parent-rated traits were 0.74, 0.65, and 0.50, respectively, and the DZ correlations were 0.47, 0.41, and 0.33. As the model fitting confirmed, the estimates were consistent with moderate heritability, some influence of the shared family environment and/or rater effects, and non-shared environmental influences and/or unreliability. The corresponding correlations for the teacher ratings, based on the shorter BPM, were smaller than parental ratings. They suggested substantial broad heritability, and no effects of the shared environment. Note that parental ratings sometimes involve contrast effects, where the twins are scored more differently than their observed behavior suggests they should be. Such effects typically also generate different phenotypic variances for MZ and DZ twins. Social, Aggression and Externalizing scales had the lowest p-values for the equal variance tests. For Attention problems, whether rated by the parents (CBCL) or teachers (BPM), MZ correlations were again consistent with substantial broad heritability including non-additive genetic variation that overwhelmed any shared environmental influence. For all other subscale scores on the CBCL, we saw correlations consistent with moderate heritability, and varying degrees of shared environmental and/or rater effects and, in some cases, evidence for non-additive genetic variance.

#### Physical and other traits (Fig. 8)

**Fig. 8:**
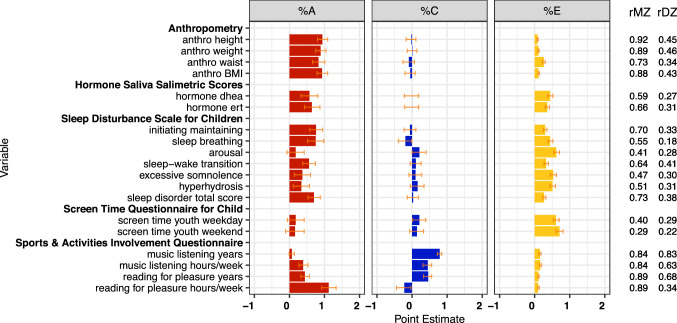
Estimates of genetic (%A), shared (%C) and specific (%E) environmental variance components and MZ and DZ twin correlations for physical and other traits

Estimated proportions of variance for physical and other traits are presented in Fig. 8. Heritability estimates for anthropometric measures (height, weight, and BMI) were around 90%. Negative estimates for shared environment suggested small contributions of non-additivity for waist circumference. Genetic factors explained ~ 60% of the variance of hormone levels. Twin correlations for the six subscales of the Sleep Disturbance Scale for Children and the sleep disorder total score were varied, resulting in *h*^*2*^ estimates ranging from 20 to 80% and high heritability for the sleep disorder total score (70%).

Individual differences in screen time in 9–10-year-old twins showed very low heritability: *h*^*2*^ = 18% for weekdays and *h*^*2*^ = 16% for weekend. For number of years listening to music, genetic contributions were also small (*h*^*2*^ = 7%), with a substantial estimate of variation associated with shared environmental factors (*c*^*2*^ = 79%). However, for number of hours per week spent listening to music, heritability was low (*h*^*2*^ = 39%) and shared environment substantial (*c*^*2*^ = 45%). The corresponding figures for ‘number of years reading for pleasure’ were *h*^*2*^ = 44% and *c*^*2*^ = 46%. Shared environmental factors explained most (*c*^*2*^ = 56%) of individual differences in number of hours per week reading for pleasure.

## Discussion

### General findings

In general, we found that univariate heritability estimates for brain, behavioral, psychiatric and anthropomorphic metrics, assessed at baseline in the ABCD study of 772 pairs of 9–10 year-old twins, are similar to those reported in previous child-focused twin studies. These consistent baseline findings were evident in a study with a rich palette of neurobehavioral, psychosocial, and brain variables, and one that is just beginning its longitudinal progression from pre-adolescence to adulthood. Thus, we can already offer variance component estimates for thousands of continuous variables, which can be consulted directly in the searchable Supplemental Online Resource at https://abcdtwinhub.shinyapps.io/baselineTwinResults, which will be expanded as new waves of data are released. The replication of univariate findings illustrates the robustness of the estimated variance components across studies, despite any methodological considerations in both ABCD data acquisition or caveats about our statistical approach. These considerations are discussed below, followed by summaries of convergence of findings with previous work in different phenotypic domains. Of note, these initial findings provide a standard reference against which comparisons can be made in multivariate and longitudinal analyses of the interrelationships between brain and behavior.

### Methodological considerations

By design, the ABCD Study® over-sampled twin pairs by incorporating four sites known for their curation of population-based samples of twins. These data provide a close match to the populations previously used for estimating genetic and environmental sources of variation across a wide variety of traits in the United States. The strategy yielded 391 MZ and 381 DZ same-sex twin pairs, with almost equal numbers of males and females, who had confirmed zygosities based on whole genome array genotyping; we expect that there will be additional pairs available in future data releases. This sample size provides sufficient power to detect additive genetic and shared environmental factors (Martin et al. 1978; Visscher et al. 2008). More concretely, with the current sample of twins with genotypically confirmed zygosity (N MZ twins = 391 pairs, N DZ twins = 381 pairs), we have 80% power to detect an additive genetic variance (VA) component explaining 20% of the variance or more, when unique environmental variance (VE) accounts for 30% or less of the remaining variance, or a VA component of 30% when VE <  = 50%. The current sample is also sufficient to detect a shared environmental variance (VC) component of 30% or more with 80% power. However, we have limited power to detect dominance genetic (VD) variance components unless they explain 50–60% of the variance (see Supplementary Fig. 1 for more details). We elected to limit our analyses to twin pairs from these four sites due to the close matching of their ascertainment methods and testing conditions. Although joint analysis with non-twins in the sample is possible, differences between population-based and school-based ascertainment methods risk biasing the results relative to other twin studies. The ABCD design permits comparison of the twin and non-twin populations’ means and variances, which do differ for some variables, but these analyses are not reported here.

Our primary focus is on the association of individual differences with additive and non-additive genetic, shared and non-shared environmental sources of variance. The analytical approach of estimating variance components without a lower bound of zero was selected to assist potential future meta-analyses of the data. The standard approach to modeling twin data from their (co)variances confounds the effects of non-additive genetic sources of variance (dominance and epistasis) with those of the shared environment. Estimates of C are therefore an aggregate of these components, with negative estimates suggesting a greater role of non-additivity than of C. Negative estimates of additive genetic variance are seen for 254 of the analyzed variables, reflecting lower MZ than DZ correlations. Results of this type seem likely to be due to sampling variation in the estimate of A when its true value is close to zero. Another possibility is that MZ and DZ variances differ appreciably, which in turn may be due to sibling interaction, or parental rating contrast effects.

The fixed effects of age, sex, race/ethnicity and site were regressed out, but not scanner (Magnetic Resonance Imaging instrument) as it was confounded with site. Random effects of scanner or site might be included if the twin and non-twin data were combined, but the non-twin data were not modeled here, due to the different ascertainment methods. Future analyses aim to include twin and non-twin data, thus allowing us to estimate how scanner type may impact variance decomposition. Similarly, we will be able to evaluate heterogeneity in variance or variance decomposition by sex, race/ethnicity and age, especially with the availability of longitudinal data.

Self-report race and ethnicity are socially-defined variables that proxy social advantages/ disadvantages and only weakly correlate with the quantitative genetic principal components based on differences in allele frequencies. In the US, Black and Hispanic Americans are at increased likelihood of exposure to a variety of environmental adversities due to institutionalized racism (e.g., housing segregation, education in under-resourced schools, victimization by the police and criminal justice systems, poverty). Thus, by regressing out the effects of sex, race/ethnicity (and correlated SES) and site in our twin models, we are partitioning the remainder of the variance in genetic and environmental sources. That said, we understand that controlling for self-identified race may not remove all influences of race on trait variance since it is an inaccurate measure. It is entirely possible that we would observe different heritability estimates across race categories, but those differences would **not** necessarily imply or suggest that different genes or biological mechanisms are the cause.

Many influential scientists in the history of behavior genetics, such as Galton, Charles Davenport, Margaret Sanger and others, were proponents of eugenics (in which those with positive traits should self-select to reproduce (positive eugenics) and those perceived as having less desirable traits discouraged or prevented from reproduction (negative eugenics)) and scientific racism, (i.e., the misuse of science, medicine, and statistics to promote the superiority of select social groups). The use of eugenics by the Nazis as a rationale for the attempted genocide in World War II stands as one of the most well-known atrocities, but many other atrocities and human rights violations have occurred and some of them were bolstered by pro-eugenics scientists. The American Eugenics Society actively campaigned to promote eugenics in US public education programs and coordinated with the Eugenics Record Office at Cold Spring Harbor Laboratory to push for the adoption of eugenic policies like forced sterilization [see the Virginia Sterilization Act of 1924 and subsequent US Supreme Court case Buck vs Bell (1927)]. The impacts of racist ideologies and policies continue to besmerch behavior genetics—which has the noble scientific goal of understanding the sources of individual differences—and are partially responsible for the dramatic underrepresentation of non-white individuals in behavioral and psychiatric genetic research cohorts (Martin et al. 2019).

Twin studies, which were first suggested by Galton, have undesirable historical associations with individuals who held socio-political beliefs that are now widely regarded as inhumane, immoral, and inherently racist. Acknowledging this history is a critical part of combating scientific racism. Twin studies can provide valuable insight regarding how environmental and genetic factors jointly contribute to the composition of phenotypic variance, and behavioral geneticists must emphasize accurate interpretations of their research and promote comprehensive and nuanced understandings of the results so that neither the public nor other scientists derive gross misunderstandings about the nature of heritability or human health. One example of emphasizing greater nuance and understanding is clarifying the relationship between social constructs like self-identified race and biological constructs like genetic ancestry/variation. These constructs are not interchangeable. The US race categories are based on US Census options, categorical, and correlated with social advantages/disadvantages and experiences of racism. Genetic ancestry/variation is continuous and based on differences in allele frequencies. Neither self-identified race nor continental ancestry assignments capture the full range of genetic variation. Given these shortcomings, we should embrace a multidimensional, continuous view of genetic variation and ancestry rather than using continental ancestry categories as suggested by a recent policy forum in Science (Lewis et al. 2022). We applaud the efforts of groups like All of Us (https://allofus.nih.gov/about/program-overview), NIH (https://www.genome.gov/about-genomics/fact-sheets/Eugenics-and-Scientific-Racism) and Pan UK Biobank (https://pan.ukbb.broadinstitute.org/) for highlighting the impacts of institutional racism on science and contribute this work and the related online resource to the ABCD Study’s own proclamation of their values, including anti-racism, inclusivity, equity, and diversity (https://abcdstudy.org/families/better-together).

### Assumptions of the twin method

Any large-scale analysis of data collected from twins should consider the assumptions of the statistical models being used. Three assumptions are most relevant: i) the phenotypic distribution of the variables; ii) the independence of the sources of variance; and iii) the equal environmental sharing assumption. First, the maximum likelihood (ML) model-fitting method used assumes that the data from twins conform to the bivariate normal distribution, albeit with different correlations for MZ and DZ pairs. This assumption was not explicitly tested, and was certainly violated at times, particularly when scale scores are derived as factor or sum scores of items where most items are rarely endorsed—such as happens with scales designed to detect clinical diagnosis or severity. Fortunately, ML is robust to failures of this type, but some underestimation of correlations is likely, compared to treating the variables as ordinal (Verhulst & Neale 2021). Such underestimation would decrease estimates of A and C or D and inflate the estimate of E.

The next two major assumptions are that there is neither covariation (rGE) nor interaction (GxE) between the variance components. The no covariance assumption would fail if, e.g., allele frequencies systematically differ between those living in low and high SES environments. Since SES covaries with race, and race differences in allele frequencies are well known (Devlin & Roeder 1999; Smith et al. 2009), this assumption is likely violated for several of the reported phenotypes. Similarly, the no interaction assumption would fail if, e.g., the effects of high SES and those of alleles associated with high cognitive ability operate synergistically to generate greater improvement than the sum of their parts. Both assumptions seem likely to have some degree of failure for some, but not all, of the traits studied. The consequences of such failures have been discussed elsewhere (Verhulst & Neale 2016). Failure of either the rGE or GxE assumption may reduce the generality of the results, require reassigning of some variance to covariance, or requiring different heritability estimates for different parts of the population distribution. Note also that absent genetic variation, neither GxE nor rGE could occur. Fortunately, features of the ABCD Study®, particularly the longitudinal assessments and collection of DNA samples for genotyping, enable testing of these assumptions in future work. A simple examination of GxE is possible by covarying MZ pair sums with their absolute differences (Jinks & Fulker 1970), and more powerful tests have been described (Molenaar et al. 2012; van der Sluis et al. 2008). Adding a relevant polygenic score to the ACE model enables estimation of covariance between A and C variance components (Dolan et al. 2021). The ongoing longitudinal assessments in ABCD will also enable direct modeling of ‘niche selection,’ where an individual’s phenotype on one occasion changes their environment (or that of their cotwin) on the next (Dolan et al. 2014).

The equal sharing of trait-relevant environmental factors (known as the equal environments assumption or EEA) is a key assumption of modeling data from a classical twin study. Many critics of the twin method ascribe findings of greater MZ than DZ correlation to violations of the assumption, citing evidence of greater environmental sharing by MZ twins than by DZ twins as sufficient evidence that it causes MZ correlations to be greater than DZ ones. However, two arguments counter this claim. First, the environments shared must be trait-relevant; if they are, then within either MZ or DZ pairs greater sharing should associate with greater phenotypic similarity. Historically, this phenomenon has rarely been observed, but improved measures of trait-relevant environmental factors are possible. Second, EEA violations upwardly bias estimates of A only when the environmental factors subject to greater sharing by MZs are not *elicited* by the twins themselves. If DZ pairs select or elicit different environments to a greater degree than do MZ pairs simply because their genotypes differ more, the attribution to the distal cause of A is appropriate. Most behavior geneticists accept that the pathways from genotype to behavior may include ‘outside the body’ environmental mediators—consistent with the concept of the extended phenotype (Dawkins & Dennett 2008; Kendler et al. 2012). Multivariate behavior genetic designs that incorporate measures of environmental factors and outcome phenotypes have great potential to dissect genetic from non-genetic sources of variation and to identify causal pathways. Modern genomic-based methods can help, too, as genotyped DZ twin pairs may be used to test the EEA in a twin study (Hwang et al. 2021). Unfortunately, very large samples of DZ pairs (perhaps ten times as many as are in ABCD) may be needed for accurate estimation of the different environmental correlations of MZ and DZ pairs—but this may be possible in future meta-analyses.

The ACE model assumes random mating in the parental generation; and in the presence of phenotypic assortative mating, may lead to underestimation of A and overestimation of C. While random mating may be a reasonable assumption for structural neuroimaging measures, it is certainly not for neurocognitive measures, a limitation that should be considered when viewing positive estimates of shared environmental variance. Conversely, unless interactions with specific environmental modifiers are explicitly modeled, variance due to A and AxC are confounded in the classical twin study design and a component of the estimated A for such neurocognitive variables may plausibly represent genotype x shared environment interaction effects (Eaves 1979, 1977).

## Substantive considerations

### Structural neuroimaging

Variance component patterns for sMRI endophenotypes were, in general, remarkably consistent with the extant literature (Blokland et al. 2012; Peper et al. 2007). As with prior studies, we observed that phenotypic variation in most global pediatric brain volumes was attributable to genetic influences. Strong genetic influences on whole brain volumes, cortical parcels and subcortical nuclei have been a relatively consistent observation across the life cycle. Shared environmental influences appeared to be relatively modest for most large volumetric endophenotypes. The observed high heritability estimates for volumetric measures makes them prime targets for molecular genetic analyses in large samples, as well as for multivariate modeling of genetically-mediated brain-behavior relationships.

We further observed substantial regional variability in the heritability of cortical thickness and surface area. Heritability patterns for cerebral surface area were subjectively similar to prior studies on newborn, childhood, adolescent, and young adult samples (Jha et al. 2018; Schmitt et al. 2019; Strike et al. 2019; Yoon et al. 2012) despite multiple methodological differences in image acquisition and image processing (e.g., FreeSurfer versus CIVET image processing pipelines). For example, consistent with prior reports (Lenroot et al. 2009; Yoon et al. 2010), genetic influences on cortical thickness were more modest relative to both surface area and volume. In general, gyral cortical thickness was more heritable than that observed within sulci. Regional heritability patterns were in many aspects similar to prior pediatric studies (Lenroot et al. 2009; Yoon et al. 2010), with the highest heritability estimates observed in peri-Sylvian, peri-Rolandic, and frontal cortex. The most heritable regions had about half of their variability explained by additive genetic factors. Posterior and inferior cerebral cortical thickness ROIs had relatively weak genetic influences. The most noteworthy discrepancy with the extant literature was a *rightward*-predominance in heritability estimates in ABCD data, particularly in the supramarginal gyrus. Studies of cortical thickness in other genetically-informative pediatric samples (NIMH, QNTS) find particularly strong genetic influences in similar regions, but with a *leftward*-predominance, i.e., preferentially influencing language centers. Leftward-predominant heritability in language centers is conceptually appealing, as it may be indicative of recent evolutionary influences in humans. The reasons for the observed discrepancies between the studies are unclear. Differences in cerebral parcellation have a relatively large influence on thickness heritability estimates and may partially explain the observed differences. There is also strong evidence that the heritability of thickness actively changes during childhood (Schmitt et al. 2014; Teeuw et al. 2019), and differences in sample age may also influence parameter estimates. The observed regional variability in thickness becomes less pronounced in adulthood, while the overall strength of genetic factors increases substantially (possibly due to fewer movement artifacts in scans from older children). Thus, neurodevelopmental considerations are important when analyzing cortical thickness data, particularly in childhood.

Unlike most prior twin neuroimaging studies, we provide heritability estimates using two distinct cortical parcellation maps, but with otherwise identical image processing, sample, and quantitative genetic models. Therefore, it may be worthwhile to briefly comment on the observed differences in heritability patterns between these two parcellations (e.g., Fig. 3). In general, heritability estimates were lower and more variable for the Destrieux atlas than the Desikan-Killiany one. The reasons for these differences are likely multiple. The Desikan-Killiany parcellation ROIs are substantially larger than Destrieux. Larger ROIs generally result in decreased measurement error (at the cost of decreased regional specificity), which in turn likely influences heritability estimates. Differences in measurement error also likely contribute to higher global heritability estimates (e.g. total brain volume) relative to regional measures. Second, ROIs for both atlases are not uniformly distributed across the cortical sheet, but rather conform to gyral and sulcal anatomy. While the Destrieux atlas attempts to distinguish between vertices within sulci versus gyri, the Desikan-Killiany does not. Given that prior studies have demonstrated co-localization between sulcal depth and the heritability of cortical thickness in older samples (Alexander-Bloch et al. 2020; Schmitt et al. 2021), proportional differences in the fraction of vertices in gyri and sulci between the two atlases may also contribute to the observed differences. The relationships between genetics, traditional brain measures (e.g. surface area), and brain shape (e.g. sulcation patterns) is a relatively unexplored research topic—particularly in children. Imaging data in ABCD will facilitate further investigations in this domain.

A major strength of ABCD is the availability of numerous imaging endophenotypes measured with standardized parcellations, facilitating neuroanatomic comparisons and multivariate analyses. In addition to more well-known measures such as thickness, we also highlight results of univariate analyses on several less commonly-investigated structural metrics. We find that sulcal depth is most heritable near the primary sulci and fissures of the brain. These sulci are the first to form during neuro-development and are the oldest evolutionarily (Armstrong et al. 1995). Lohmann et al. observed that deeper, ontologically older sulci were more alike in MZ twins relative to unrelated individuals, suggesting that genetic influences were stronger than those of secondary and tertiary sulci (Lohmann et al. 1999). These observations have subsequently been supported by several additional studies using more traditional quantitative genetic analysis and numerous measures of sulcation (Le Guen et al. 2018). Genetically-informative studies on T1/T2 image contrast are more limited. Although T1 contrast has previously been reported as a heritable phenotype in older adults (Panizzon et al. 2012), here we report similar findings in a pediatric sample. Furthermore, to our knowledge this study represents the first to observe similar patterns for T2 contrast, but with substantially increased genetic signal relative to T1 contrast. Heritability maps for both T1 and T2 contrast are very similar to those based on cortical myelination (as defined by T1/T2 ratio), with a pattern of greater heritability in posterior regions (Schmitt et al. 2020). In humans, more evolutionarily expanded regions of the cortex (relative to other primates) tend to be less myelinated than more ontologically stable areas (Glasser et al. 2014).

There is pronounced development of white matter during adolescence. The most commonly observed patterns in dMRI studies are observations of age- and puberty-related maturational increases in FA and decreases in MD across major fiber tracts (Lebel & Beaulieu 2011; Tamnes et al. 2018). Developmental changes appear steepest during childhood with decelerations in adolescence followed by a plateau in early-to-mid adulthood (Tamnes et al. 2018). Sex differences have been observed with some suggestion of steeper changes in males relative to females, perhaps due to the influence of gonadal hormones on neurodevelopmental processes (Herting et al. 2012; Simmonds et al. 2014). Thus, it would not be surprising to find age-related differences in heritability patterns moderated by other characteristics. However, prior studies have consistently reported moderate to high heritabilities of white matter metrics such as FA and MD within major fiber tracts that, when aggregated, vary relatively little from childhood to adulthood (Chiang et al. 2011; Gustavson et al. 2019; Kochunov et al. 2015; Zhao et al. 2019). Diffusion MRI findings from the current study are broadly consistent with those from previous studies, which is notable considering that ABCD is a multi-site study that includes three scanner platforms, varying acquisition sequences specific to each platform, and a challenging 9–10-year-old sample. Such variations might be expected to impact the quality of dMRI data and reliability estimates (Tamnes et al. 2018). Our replication of prior dMRI heritability findings is reassuring. Similar to what was observed for cortical thickness, heritability estimates were often higher for right hemisphere tracts and particularly for FA. The reasons for this patterning are unclear given that the right hemisphere is non-dominant for most individuals in the population. Although not explored here, others have found modulatory effects of sex, IQ, and SES on FA and MD heritabilities (Chiang et al. 2011; Kochunov et al. 2015). This area merits further exploration within the ABCD dataset. The lack of shared environmental influences on dMRI metrics that we observed is consistent with other reports (Gustavson et al. 2019), as is the finding that unshared environment exerts a moderate influence on white matter microstructure.

### Neurocognition

The ABCD cognitive battery was designed with several principles in mind (Luciana et al. 2018). Measures were selected to be neuroscientifically informed, psychometrically sound, and relevant to substance use outcomes. The battery had to be amenable to longitudinal assessment with minimal practice effects. Tasks were selected to be sensitive to developmental effects with minimal floor and ceiling effects. In addition, the psychometric integrity of selected measures is crucial to reliable measurement, so selection criteria emphasized that measures must show adequate reliability and validity. The battery was computerized to promote standardization and facilitate multi-site administration. Measures that require minimal training were used to reduce staff burden and likelihood of measurement error. The end result is a set of measures that do not necessarily approximate, in length or breadth, those that have been used in other behavior genetic studies of cognition. For instance, a full IQ battery was not administered, and measures of discrete abilities such as EF were truncated versions of measures.

As an alternative to a full IQ battery, composite indices of crystallized and fluid abilities were derived from the NIH Toolbox. These measures show patterns of genetic influence that cohere with other studies of children and adolescents. Findings for discrete abilities such as EF, learning, memory and spatial ability are less clear cut and more variable, which is true in the literature as a whole. There are relatively few studies in children against which to benchmark the current findings. However, the low heritability for the principal component that reflects EF contradicts some studies, in which variance common to EF tasks was 100% heritable (Engelhardt et al. 2015; Friedman et al. 2008) but is consistent with others that estimated near zero heritability for the Flanker Task (Stins et al. 2004). Note that all EF tests in ABCD are timed tasks, and the influence of processing speed remains to be seen.

The observed heritabilities for measures of spatial ability in ABCD are low relative to the published literature. A meta-analysis of visuospatial reasoning studies (King et al. 2019) in twins found considerably higher heritability estimates for Matrix Reasoning (58%) and Mental Rotation (65%). Across visuospatial domains, heritabilities were close to 47% for children. A possible reason for the difference is that the ACE model may be inappropriate for ABCD cognitive data. However, the model we used allows variance component estimates to be negative, so substantial non-additive genetic variance would emerge as a negative estimate of C, unless C itself was also sizable. We do observe negative estimates of C for many measures. In the event that the shared environment has near zero effect, minus twice the negative estimate of C could be interpreted as genetic non-additivity, and added to the revised estimate of A (A’ = A + 3C) to obtain an estimate of broad heritability. One solution to the problem of confounded parameters is to improve the research design to resolve them, for example by adding different types of relatives, such as half-siblings or adoptees or extended pedigrees (Truett et al. 1994). However, in the present case, many estimates of E are high (driven by relatively low MZ correlations) and would not decline by adding different types of relatives.

It is possible that the proportion of measurement error in some of the assessments is large; if it is, the test–retest correlations across waves should also be low, which is not uniformly the case. Prior assessments of test–retest correlations for the NIH Toolbox have been few but suggest highly stable measures (Gershon et al. 2014; Gershon et al. 2013; Mungas et al. 2014). For instance, in validation testing with children and adolescents (Zelazo et al. 2013), the Flanker task showed excellent test–retest reliability (ICC = 0.92). For the RAVLT test–retest reliability is lower (r-values ~ 0.60–0.70; (van den Burg & Kingma 1999). The ‘gold standard’ Matrix Reasoning measure has a retest correlation of r = 0.78 for 9–10-year-old children based on the standardization sample (Wechsler 2014). For the Little Man task suitable reliability data seem not to exist, but test–retest correlations of EF appear to be in the 0.70–0.90 range (Beck et al. 2011). A recent report on longitudinal test–retest stability of individual differences in ABCD participants’ baseline to year 2 data [Anokhin, (under review)] indicates that retest stability ranges from fair (Flanker test: r = 0.43) to excellent (Crystallized Intelligence composite: r = 0.82). ABCD’s observed stability estimates for the NIH TB are highly consistent with a three-year longitudinal study of NIH TB in youth aged 9 to 15 (Taylor et al. 2020).

Heritabilities of performance on the NIH Toolbox seem roughly on par with the results in adults (Pinto et al. 2020), where general aggregate cognition metrics showed heritabilities around 50%, with individual task or cognitive factor score heritabilities generally lower. The findings are consistent with expectations for the composite measures of fluid and crystallized ability. However, when discrete tasks are examined, unexpected but intriguing patterns emerge, primarily for measures of executive function such as inhibitory control. Studies reporting on the heritabilities of discrete executive functions, based on twin samples, are rare and usually based on small sample sizes.

### Childhood psychopathology

For the CBCL parental ratings of their children’s behavior, and for teacher ratings of the children, the patterns of twin resemblance were largely as expected for our 9–10-year-olds at this baseline assessment. MZ correlations in all instances exceeded those of DZ pairs and did so to an extent consistent with moderate heritability, some evidence of shared environmental influences for parental ratings, but no evidence of shared environmental influences based on teacher ratings. For attention problems, heritability may be higher than for other behaviors, though multivariate analyses would be required to compare them statistically. The difference in variance components for ratings by parents and teachers may reflect rater effects, inflated shared environmental variance for parental ratings (Hewitt et al. 1992), or that problem behavior is situation specific and less correlated for twins or siblings in the school situation than in the home (Haberstick et al. 2005, 2006). Furthermore, there may exist rater or sibling interaction effects on the Sociability, Aggression, and Externalizing scales. The univariate analyses of these ABCD Study® baseline assessments are consistent with the patterns of genetic and environmental influences on problem behaviors observed in the previous behavior genetic literature. The project will provide a robust basis for exploring the development of problem behavior in different situations. We have not yet addressed differences between boys and girls.

### Physical and other traits

The heritability estimates for height, weight, and BMI were high to very high and in line with those from previous studies. However, rather than small shared environmental contributions to anthropometric measures, the negative estimates for C suggested non-additive influences. For hormone levels, current heritability estimates appeared on par with previously reported estimates, and consistent with genetic influences explaining a significant proportion of the variation. Genetic factors associated with most of the variation in the items of the Sleep Disturbance Scale for Children, with heritability estimates higher than those reported on sleep characteristics in adolescence. For a number of measures, such as screen time, and various recreational activities such as listening to music and reading, the ABCD Study® is generating the first estimates of the role of genetic and environmental factors to the observed variation among 9- and 10-year-old twins.

#### Supplemental online resources

The supplemental online resources (https://abcdtwinhub.shinyapps.io/baselineTwinResults) include a searchable data dashboard that displays the twin model results for all tested baseline continuous variables. Users can adjust the output to display the twin correlations by zygosity and sex, and the standardized and unstandardized parameter estimates of the ACE model either for the full sample or by sex, or both. Due to the size of the data and a desire to minimize loading time, the results have been organized by research domain, which users can select at the top left of the screen in the section “Select a Variable Category”. The dashboard also hosts the figures from this manuscript and background content salient to the ABCD Study and twin methods. A link to three-dimensional visualizations of parameter estimates for brain regions can be viewed using either the Desikan-Killiany or the Destrieux atlas at https://schmittje.shinyapps.io/ABCD_brainviz (linked in the ABCD dashboard in the section entitled “3D Brain Images”). To launch the application, users select the parameter and participant subset (i.e., all participants, males only, or females only) of interest, a brain atlas for plotting, and click “Render Plot”. A concise overview of the abbreviations is included as a reference under “Additional Information”. Users should note that the Destrieux atlas takes considerably longer to render than the Desikan-Killiany atlas, owing to the greater level of detail in the former.

## Future opportunities

These analyses of 14,500 variables using the twin study site ABCD data are only an initial exploration of the sources of variation in the 3.0 dataset. There is enormous potential for future analyses of many types. Within the twin sites’ data, sex or race/ethnicity and SES differences in means and sources of variation could be estimated, although the sample size is modest for comparison of variance components. Twin versus non-twin comparisons can be made, both within the four twin sites and across all sites. The fact that non-twins also are assessed at the four twin sites is another useful feature that will help to distinguish twin versus non-twin differences from site effects.

The ABCD dataset contains around 50,000 additional variables—ordinal data or continuous measures taken as part of functional MRI tests—that are not discussed in this article. The ordinal data will be addressed in a future paper, using Bayesian methods to estimate site variance together with parameters of the variance components models used here. Indeed, reanalysis using the entire ABCD dataset promises to be interesting, since there appear to be some systematic differences between the population-based sampling in twin sites and the school-based sampling used by the majority of ABCD Study sites. For example, the variance of height differs between twins and non-twins, such that a combined analysis would yield a lower heritability estimate and some evidence of shared environmental factors. However, the optimal joint analysis of the twin site and non-twin site data has yet to be determined. While it is possible that lower estimates of genetic variance components better represent the population as a whole, the population-based sampling used by the four twin sites may provide a more accurate estimate of population variance.

Variance components of task-fMRI activations are not discussed in this article for two main reasons. First, we observed very low twin correlations for many of these measures, and preliminary analyses of the longitudinal data suggest low test–retest reliability (Kennedy et al. 2022). The greater noise introduced by frequent incidence and more severe head motion in 9–10 year-old children will most certainly complicate modeling of variance components in task fMRI. A related limitation is the potential for segmentation errors in Freesurfer, shown to be increased in motion-degraded structural scans (Reuter et al. 2015). It will be important to analyze change in variance components of structural MRI metrics with development in future releases controlling for likely reduction in head-motion as children age. Low correlations may also arise from contrast-based derived task fMRI data. When two traits’ variance components correlate highly the genetic variance of the difference scores will be close to zero. Unfortunately, raw scores from which differences are derived are not included in the ABCD 3.0 data release. The Supplemental Online Resource includes analyses of all available continuous measures, including functional neuroimaging.

Perhaps most exciting is the potential for multivariate analyses. A major part of the rationale for including the twin data is that they help to test hypotheses about direction of causation (Heath et al. 1993). Opportunities to evaluate the moderating effects of sex, race/ethnicity/ancestry, SES and other potential covariates are abundant. This capability improves with both longitudinal assessments and genetic marker data, both of which are integral parts of the ABCD Study®. The complexity and diversity of measures in ABCD will support not only brain-wide association scans but also genome-wide association studies once the genotype data is available. Moreover, as the breadth and depth of the longitudinal data increases, so too will opportunities to apply and develop new analytic approaches to characterize developmental trajectories, predict outcomes, and investigate the interplay of genetic, neurological, social, behavioral, and cognitive factors.

## Supplementary Information

Below is the link to the electronic supplementary material.

Supplementary file1 (PDF 18 kb)

Supplementary file2 (DOCX 244 kb)

## Data Availability

ABCD Study data are publicly available.

## Abbreviations

A: Additive genetic variance
ABCD: Adolescent Brain Cognitive Development™
ACE: Model with additive genetic, shared and unique environmental sources of variance
ADE: Model with additive and dominance genetic, and unique environmental sources of variance
BMI: Body mass index
BPM: Brief Problem Monitor
C: Shared or common environment
CB: Cognitive Battery
CC: Corpus callosum
CTS: Classical twin study
CUB: University of Colorado Boulder
CBCL: Child Behavior Checklist
D: Dominance genetic variance
dMRI: Diffusion magnetic resonance imaging
DTI: Diffusion tensor imaging
DZ: Dizygotic
E: Specific, unique or individual environment
EEA: Equal environment’s assumption
EF: Executive function
FA: Fractional anisotropy
g: General cognitive ability
GxE: Genotype by environment interaction
IQ: Intelligence quotient
LMT: The Little Man Task
MD: Mean diffusivity
ML: Maximum likelihood
MRI: Magnetic resonance imaging
MZ: Monozygotic
NDA: National Data Archive
NIHTB: National Institutes of Health Toolbox
RAVLT: Rey Auditory Verbal Learning Test
rDZ(s): Dizygotic twin correlation(s)
rGE: Genotype environment correlation
rMZ(s): Monozygotic twin correlation(s)
ROIs: Regions of interest
SDSC: Sleep Disturbance Scale for Children
SEM: Structural equation modeling
SES: Socio-economic status
sMRI: Structural magnetic resonance imaging
UMN: University of Minnesota
VCU: Virginia Commonwealth University
WUSTL: Washington University at St. Louis
WISC-V: Wechsler Intelligence Test for Children V

## Code Availability

Code is available in the Supplementary Materials.
