# Supplementary material for: Genetic and Environmental Variation in Continuous Phenotypes in the ABCD Study®
Source: Behav Genet. 2022 Nov 10;53(1):1–24. doi: 10.1007/s10519-022-10123-w (PMC9823057; doi:10.1007/s10519-022-10123-w)
Supplement: Supplementary file 1 — Supplementary file1 (PDF 18 kb) [file 10519_2022_10123_MOESM1_ESM.pdf]

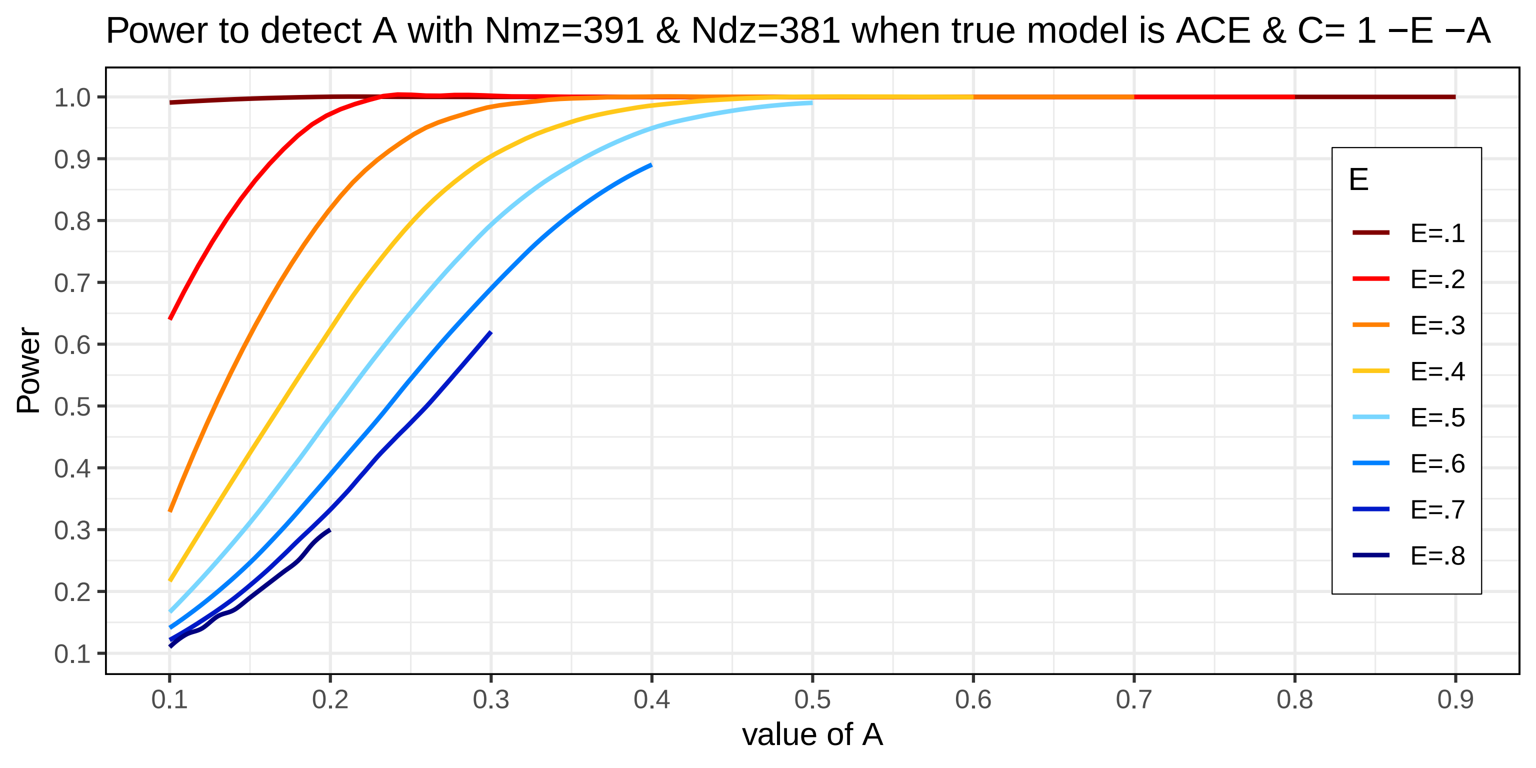
Power to detect A with Nmz=391 & Ndz=381 when true model is ACE & C= 1 −E −A
Power
1.0
0.9
0.8
0.7
0.6
0.5
0.4
0.3
0.2
0.1
0.1
0.2
0.3
0.4
0.5
0.6
0.7
0.8
0.9
value of A
E
E=.1
E=.2
E=.3
E=.4
E=.5
E=.6
E=.7
E=.8

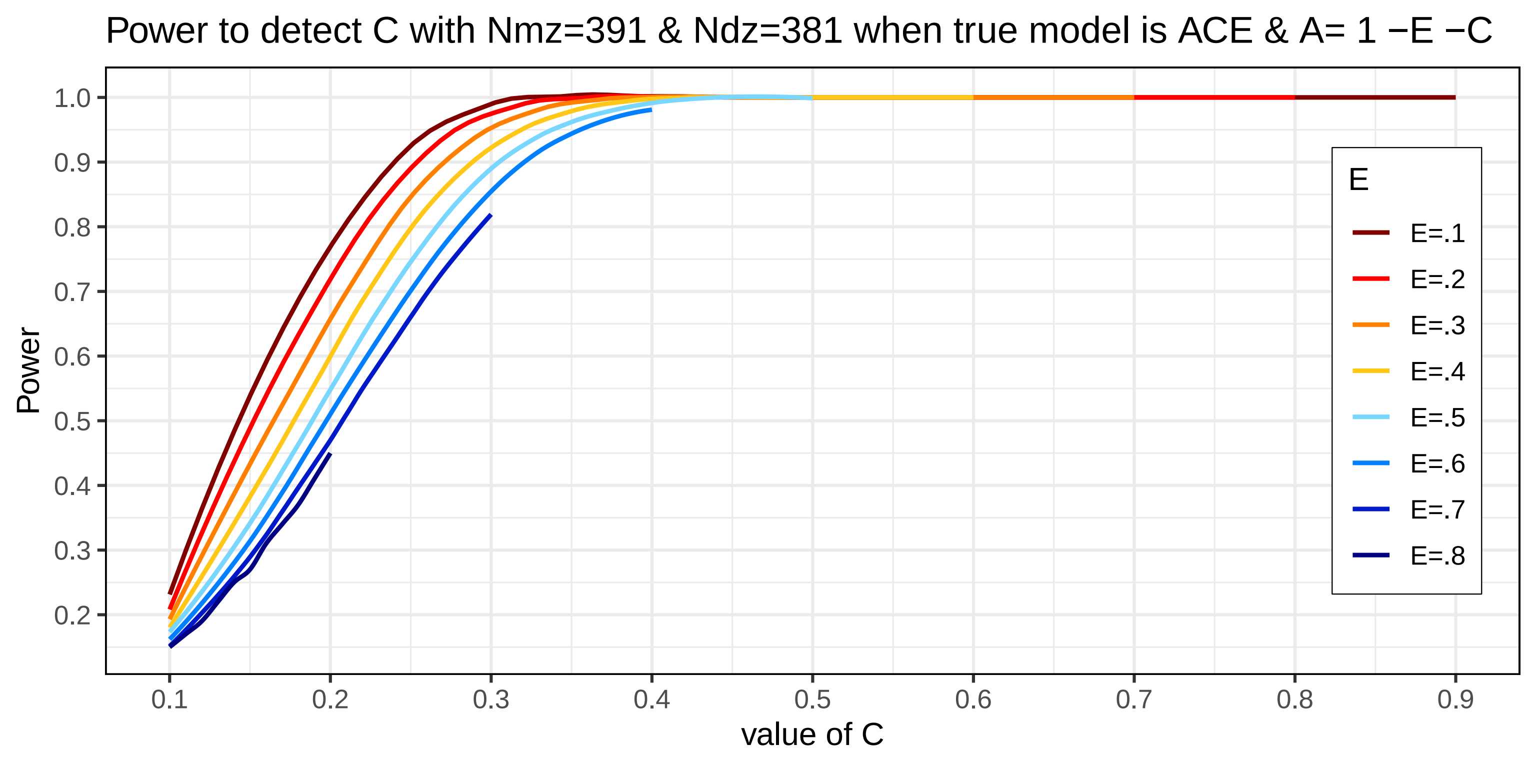

Power to detect C with Nmz=391 & Ndz=381 when true model is ACE & A= 1 −E −C
Power
1.0
0.9
0.8
0.7
0.6
0.5
0.4
0.3
0.2
0.1
0.2
0.3
0.4
0.5
0.6
0.7
0.8
0.9
value of C
E
E=.1
E=.2
E=.3
E=.4
E=.5
E=.6
E=.7
E=.8

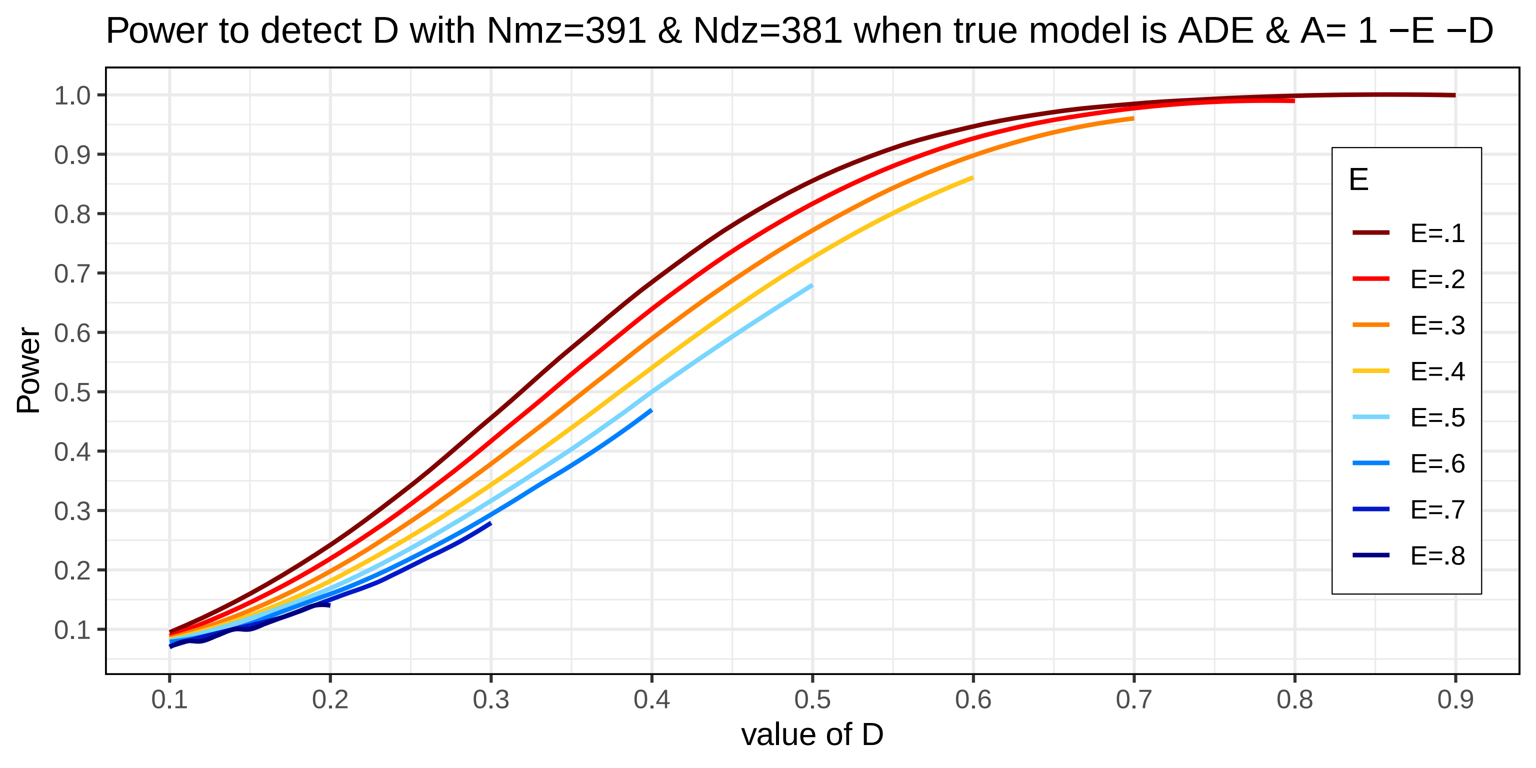
Power to detect D with Nmz=391 & Ndz=381 when true model is ADE & A= 1 −E −D
Power
1.0
0.9
0.8
0.7
0.6
0.5
0.4
0.3
0.2
0.1
0.1
0.2
0.3
0.4
0.5
0.6
0.7
0.8
0.9
value of D
E
E=.1
E=.2
E=.3
E=.4
E=.5
E=.6
E=.7
E=.8
