# Supplementary material for: Genetic and Environmental Variation in Continuous Phenotypes in the ABCD Study®
Source: Behav Genet. 2022 Nov 10;53(1):1–24. doi: 10.1007/s10519-022-10123-w (PMC9823057; doi:10.1007/s10519-022-10123-w)
Supplement: Supplementary file 2 — Supplementary file2 (DOCX 244 kb) [file 10519_2022_10123_MOESM2_ESM.docx]

Genetic and environmental variation in continuous phenotypes in the ABCD Study^®^

Supplemental Materials

[Measures 2](#_Toc109416682)

[1. ABCD Baseline Structural Neuroimaging Battery 2](#_Toc109416683)

[2. ABCD Baseline Neurocognitive Battery 2](#_Toc109416684)

[3. ABCD Childhood Psychopathology Battery 4](#_Toc109416685)

[4. ABCD Physical and Other Traits 5](#_Toc109416686)

[References 6](#_Toc109416687)

[Prior Genetically-Informative Studies 10](#_Toc109416688)

[1. Structural Neuroimaging 10](#_Toc109416689)

[1.1. Regional Measures of Morphometry 10](#_Toc109416690)

[1.2. Anatomical Measures of Connectivity 12](#_Toc109416691)

[2. Neurocognition 13](#_Toc109416692)

[3. Child Psychopathology 15](#_Toc109416693)

[4. Physical and Other Traits 16](#_Toc109416694)

[References 18](#_Toc109416695)

## Measures

The NIMH National Data Archive (NDA) ABCD 3.0 publicly available database was used for this analysis. All continuous variables as well as those with more than twelve categories were included in the analyses, totaling 53,158 variables. We focus on 13,532 variables, covering structural neuroimaging, neurocognition, childhood psychopathology and physical, hormonal and sleep measures. Tabular results in Supplemental Online Information (https://abcdtwinhub.shinyapps.io/baselineTwinResults/#section-tabular-results) include results from *all* tabulated (i.e. pre-calculated) continuously distributed variables in the open access dataset, which are described in ABCD Data Release documentation, including results discussed here on 13,532 variables, and an additional 39,626 task MRI related variables.

### 1. ABCD Baseline Structural Neuroimaging Battery

Details of the ABCD neuroimaging acquisition sequences and processing streams have been presented elsewhere (Casey et al., 2018; Hagler et al., 2019). The ABCD imaging protocol was harmonized across data collection sites for three 3T scanning systems (Siemens Prisma, Philips, General Electric 750), all of which utilized standard adult-size multi-channel head coils and multiband echo planar imaging (EPI) acquisitions. The diffusion MRI acquisition had high resolution (1.7 mm isotropic voxels) and utilized multiband EPI (Hagler et al., 2019). The scanning sequences that yield structural data (Casey et al., 2018) include a localizer, T-1 weighted scan, diffusion tensor imaging (DTI), and T-2 weighted scans. Real-time motion detection and correction during acquisition are implemented by customized hardware and software. Imaging parameters were harmonized as much as possible between scanner manufacturers.

### 2. ABCD Baseline Neurocognitive Battery

The NIH Toolbox® –Cognition battery (herein referred to as “the Toolbox”) was administered and includes seven tasks that measure episodic memory, executive function, attention, working memory, processing speed, and language abilities. We report here on the uncorrected Toolbox scores. Composite indices of fluid and crystallized reasoning are also derived (Bleck et al., 2013; Gershon, Wagster, et al., 2013; Hodes et al., 2013). The neurocognitive battery has been more extensively described elsewhere (Luciana et al., 2018; Thompson et al., 2019). Here, we briefly describe each task.

The Toolbox Picture Vocabulary Task® (Gershon et al., 2014; Gershon, Slotkin, et al., 2013) is a variant on the Peabody Picture Vocabulary Test (PPTV) and measures language as well as verbal intellect. Children hear audio files of words and are shown four pictures in a square, one of which depicts the concept, idea or object referenced by the auditorily presented words. The child is asked to touch the picture that matches the word. The task uses computerized adaptive testing (CAT) to ensure appropriate item difficulty in an efficient format.

The Toolbox Oral Reading Recognition Task^®^ is a reading test that asks individuals to pronounce single letters or words presented in the middle of the iPad screen (Gershon et al., 2014; Gershon, Slotkin, et al., 2013) and measures exposure to language materials as well as the cognitive skills involved in reading. The oral reading task also uses CAT with items calibrated using item response theory to ensure appropriate item difficulty in an efficient format.

The Toolbox Pattern Comparison Processing Speed Test^®^ (Carlozzi et al., 2015; Carlozzi et al., 2014; Carlozzi et al., 2013), was modeled on the Pattern Comparison Task developed by Salthouse (Salthouse et al., 1991) and is a measure of rapid visual processing. Children are shown two pictures and asked to indicate by touch input whether the pictures are the same or not. The score is based on how many items they are able to complete correctly in a specific amount of time.

The Toolbox List Sorting Working Memory Test® is a variant of the letter-number sequencing test (Gold et al., 1997) that uses pictures rather than words or letters (Tulsky et al., 2014; Tulsky et al., 2013). The basic task is to use working memory to sequence task stimuli based on category membership and perceptual characteristics.

The Toolbox Picture Sequence Memory Test® was modeled after memory tests asking children to imitate a sequence of actions with props developed by Bauer and colleagues. Participants are presented with a series of fifteen pictures depicting activities or events that could occur in a particular setting (e.g., working on a farm) (Bauer et al., 2013; Dikmen et al., 2014). They must reproduce the sequence as it was presented. The task is not timed.

The Toolbox Flanker Task®, a variant of the Eriksen Flanker task (Eriksen & Eriksen, 1974), was adapted from the Attention Network Task (Fan et al., 2002; Rueda et al., 2004). It assesses the degree to which participants’ responses are influenced by whether the surround stimuli and the target are congruent or incongruent.

The Toolbox Dimensional Change Card Sort Task® was based on the work of Zelazo and colleagues (Zelazo, 2006) and measures cognitive flexibility. In this task, children are presented with objects at the bottom of the screen (e.g., white rabbit, green boat). The participant is asked to sort a third object presented in the middle of the screen by either color or shape to match one of the two objects on the bottom of the screen (Zelazo et al., 2014; Zelazo et al., 2013). After initial practice, they first do a block of trials sorting on one of the dimensions, then they do a block of trials in which they are told to switch to the other dimension. Lastly, they do a block of trials where they alternate in pseudorandom order between sorting on shape versus color.

Because the Toolbox measures do not employ delayed recall or recognition trials as part of their memory assessments, the Rey Auditory Verbal Learning Test (RAVLT), a widely used test of auditory learning and memory, was implemented for this purpose. Participants listen to and recall a list of 15 unrelated words over five learning trials. Following initial learning of the list, a distractor list of 15 words is presented, and the participant is asked to recall as many words from this second list as he/she is able. Next, recall of the initially learned list is assessed. To assess longer-term retention of the list, following a 30-minute delay, recall is again assessed. Through a collaboration with Pearson Clinical Assessments, ABCD created a customized automated version that built upon Pearson’s Q-interactive automated testing platform (http://www.helloq.com/home.html).

An automated version of the Matrix Reasoning subtest from the Wechsler Intelligence Test for Children-V (WISC-V)(Wechsler, 2014) was administered in standardized format using automated technology (Q-interactive (Daniel et al., 2014)).

The Little Man Task (LMT) (Acker & Acker, 1982) was utilized to engage visual-spatial processing, specifically mental rotation with varying degrees of difficulty. The task involves the presentation of a rudimentary male figure holding a briefcase in one hand in the middle of the screen. The figure may appear in one of four positions; right side up versus upside down and either facing the respondent or with his back to the respondent. The briefcase may be in either the right or left hand. Using one of two buttons, respondents indicate which hand is holding the briefcase. Accuracy and reaction time are measured for each trial, and an efficiency score is calculated as a function of both.

Members of our group have published a principal components analysis of the battery (Thompson et al., 2019), including intercorrelations among measures. The three principal components were derived from performance on the NIH Toolbox, RAVLT, and LMT measures: they reflect General Ability, Executive Function, and Learning/Memory, respectively.

### 3. ABCD Childhood Psychopathology Battery

As noted in the introduction, in the baseline assessment of 9- and 10-year-old children, parents rate their children’s externalizing and internalizing behavior using the Child Behavior Checklist (CBCL: (Achenbach & Edelbrock, 1981, 1983)). The 112-item checklist yields an overall Externalizing behavior score and an overall Internalizing behavior score, as well as subscale scores. Subscale scores include DSM5-oriented ADHD, Anxiety Disorder, Conduct Disorder, Depression, Oppositional behavior, and Somatic Problems. Subscale syndrome scores include those for aggressive behavior, anxiety/depression, attention, rule breaking, social difficulties, somatic problems, thought problems, and withdrawn depression, as well as other problems and an overall total problem score. Additional scale scores are available for Sluggish Cognitive Tempo, Obsessive-Compulsive Problems, and Stress. Each scale score is available as a raw score summation of 0 (Not true), 1 (Somewhat/sometimes), 2 (Very true/Often true) responses, and as a transformed t-score used more commonly by clinicians to provide interpretive guidance. We would expect raw scores and t-scores to behave similarly in most applications or analyses and report results here for raw scores. For the teacher ratings (BPM: (Achenbach et al., 2011)), the 18-item checklist was scored for the overall internalizing and externalizing scales, attention problems, and a total problem score.

### 4. ABCD Physical and Other Traits

We report here on physical and other traits that are measured as continuous variables in ABCD. Standardized measurements following CDC guidelines were used to monitor health, obesity and growth and physical development (CDC (Division of Nutrition), 2016), including height, weight, body mass index, and waist circumference. Pubertal hormones were assessed through the collection of a single salivary biospecimen from which salimetric scores for DHEA, testosterone and estradiol were derived. The Sleep Disturbance Scale for Children (SDSC) (Bruni et al., 1996) comprehensively screens for a variety of sleep disturbance types using a 26-item Likert-type rating scale administered to a parent. Six scales (disorders of initiating and maintaining sleep, sleep breathing disorders, disorders of arousal or nightmares, sleep wake transition disorders, disorders of excessive somnolence, and sleep hyperhidrosis) and an overall sleep-wake disturbance symptom severity score were derived. A measure of visual media use (Sharif et al., 2010) included two questions on the overall amount of time that the youth spends using visual media, one about a typical weekday and one about a typical weekend day. Extracurricular activities, including sports, music related activities, or hobbies, were assessed with the parent-reported Sports and Activities Involvement Questionnaire (Huppertz et al., 2016). In this report, we included continuous measures of years and hours per week of music listening and reading for pleasure. See (Barch et al., 2018) for more details on measure.

### References

Achenbach, T. M., & Edelbrock, C. S. (1981). Behavioral problems and competencies re­ ported by parents of normal and disturbed children aged four through sixteen. *Monogr. Soc. Res. Child Dev., 46*.

Achenbach, T. M., & Edelbrock, C. S. (1983). *Manual for the Child Behavior Checklist and Revised Child Behavior Profile.* Dep. Psychiatry, Univ. Vermont.

Achenbach, T. M., McConaughy, S. H., Ivanova, M. Y., & Rescorla, L. A. (2011). *Manuel for the ASEBA Brief Problem Monitor*. Research Center for Children, Youth, and Families, University of Vermont.

Acker, W., & Acker, W. (1982). *Bexley Maudsley Automated Processing Screening and Bexley Maudsley Category Sorting Test Manual.* NFER-Nelson Publishing.

Barch, D. M., Albaugh, M. D., Avenevoli, S., Chang, L., Clark, D. B., Glantz, M. D., Hudziak, J. J., Jernigan, T. L., Tapert, S. F., Yurgelun-Todd, D., Alia-Klein, N., Potter, A. S., Paulus, M. P., Prouty, D., Zucker, R. A., & Sher, K. J. (2018, Aug). Demographic, physical and mental health assessments in the adolescent brain and cognitive development study: Rationale and description. *Dev Cogn Neurosci, 32*, 55-66. <https://doi.org/10.1016/j.dcn.2017.10.010>

Bauer, P. J., Dikmen, S. S., Heaton, R. K., Mungas, D., Slotkin, J., & Beaumont, J. L. (2013, Aug). III. NIH Toolbox Cognition Battery (CB): measuring episodic memory. *Monogr Soc Res Child Dev, 78*(4), 34-48. <https://doi.org/10.1111/mono.12033>

Bleck, T. P., Nowinski, C. J., Gershon, R., & Koroshetz, W. J. (2013, Mar 5). What is the NIH toolbox, and what will it mean to neurology? *Neurology, 80*(10), 874-875. <https://doi.org/10.1212/WNL.0b013e3182872ea0>

Bruni, O., Ottaviano, S., Guidetti, V., Romoli, M., Innocenzi, M., Cortesi, F., & Giannotti, F. (1996, Dec). The Sleep Disturbance Scale for Children (SDSC). Construction and validation of an instrument to evaluate sleep disturbances in childhood and adolescence. *J Sleep Res, 5*(4), 251-261. <https://doi.org/10.1111/j.1365-2869.1996.00251.x>

Carlozzi, N. E., Beaumont, J. L., Tulsky, D. S., & Gershon, R. C. (2015, Aug). The NIH Toolbox Pattern Comparison Processing Speed Test: Normative Data. *Arch Clin Neuropsychol, 30*(5), 359-368. <https://doi.org/10.1093/arclin/acv031>

Carlozzi, N. E., Tulsky, D. S., Chiaravalloti, N. D., Beaumont, J. L., Weintraub, S., Conway, K., & Gershon, R. C. (2014, Jul). NIH Toolbox Cognitive Battery (NIHTB-CB): the NIHTB Pattern Comparison Processing Speed Test. *J Int Neuropsychol Soc, 20*(6), 630-641. <https://doi.org/10.1017/S1355617714000319>

Carlozzi, N. E., Tulsky, D. S., Kail, R. V., & Beaumont, J. L. (2013, Aug). VI. NIH Toolbox Cognition Battery (CB): measuring processing speed. *Monogr Soc Res Child Dev, 78*(4), 88-102. <https://doi.org/10.1111/mono.12036>

Casey, B. J., Cannonier, T., Conley, M. I., Cohen, A. O., Barch, D. M., Heitzeg, M. M., Soules, M. E., Teslovich, T., Dellarco, D. V., Garavan, H., Orr, C. A., Wager, T. D., Banich, M. T., Speer, N. K., Sutherland, M. T., Riedel, M. C., Dick, A. S., Bjork, J. M., Thomas, K. M., Chaarani, B., Mejia, M. H., Hagler, D. J., Jr., Daniela Cornejo, M., Sicat, C. S., Harms, M. P., Dosenbach, N. U. F., Rosenberg, M., Earl, E., Bartsch, H., Watts, R., Polimeni, J. R., Kuperman, J. M., Fair, D. A., Dale, A. M., & Workgroup, A. I. A. (2018, Aug). The Adolescent Brain Cognitive Development (ABCD) study: Imaging acquisition across 21 sites. *Dev Cogn Neurosci, 32*, 43-54. <https://doi.org/10.1016/j.dcn.2018.03.001>

CDC (Division of Nutrition). (2016). Anthropometry Procedures Manual. <https://wwwn.cdc.gov/Nchs/Data/Nhanes/2015-2016/Manuals/2016_Anthropometry_Procedures_Manual.pdf>.

Daniel, M. H., Wahlstrom, D., & Zhang, O. (2014). *Equivalence of Q-interactive® and Paper Administrations of Cognitive Tasks: WISC®–V: Q-Interactive* (Technical Report 8. <http://www.helloq.com/content/dam/ped/ani/us/helloq/media/Technical-Report_WISC-V_092514.pdf>., Issue.

Dikmen, S. S., Bauer, P. J., Weintraub, S., Mungas, D., Slotkin, J., Beaumont, J. L., Gershon, R., Temkin, N. R., & Heaton, R. K. (2014, Jul). Measuring episodic memory across the lifespan: NIH Toolbox Picture Sequence Memory Test. *J Int Neuropsychol Soc, 20*(6), 611-619. <https://doi.org/10.1017/S1355617714000460>

Eriksen, B. A., & Eriksen, C. W. (1974). Effects of noise letters upon the identification of a target letter in a nonsearch task. *Perception & Psychophysics, 16*(1), 143-149.

Fan, J., McCandliss, B. D., Sommer, T., Raz, A., & Posner, M. I. (2002, Apr 1). Testing the efficiency and independence of attentional networks. *J Cogn Neurosci, 14*(3), 340-347. <https://doi.org/10.1162/089892902317361886>

Gershon, R. C., Cook, K. F., Mungas, D., Manly, J. J., Slotkin, J., Beaumont, J. L., & Weintraub, S. (2014, Jul). Language measures of the NIH Toolbox Cognition Battery. *J Int Neuropsychol Soc, 20*(6), 642-651. <https://doi.org/10.1017/S1355617714000411>

Gershon, R. C., Slotkin, J., Manly, J. J., Blitz, D. L., Beaumont, J. L., Schnipke, D., Wallner-Allen, K., Golinkoff, R. M., Gleason, J. B., Hirsh-Pasek, K., Adams, M. J., & Weintraub, S. (2013, Aug). IV. NIH Toolbox Cognition Battery (CB): measuring language (vocabulary comprehension and reading decoding). *Monogr Soc Res Child Dev, 78*(4), 49-69. <https://doi.org/10.1111/mono.12034>

Gershon, R. C., Wagster, M. V., Hendrie, H. C., Fox, N. A., Cook, K. F., & Nowinski, C. J. (2013, Mar 12). NIH toolbox for assessment of neurological and behavioral function. *Neurology, 80*(11 Suppl 3), S2-6. <https://doi.org/10.1212/WNL.0b013e3182872e5f>

Gold, J. M., Carpenter, C., Randolph, C., Goldberg, T. E., & Weinberger, D. R. (1997, Feb). Auditory working memory and Wisconsin Card Sorting Test performance in schizophrenia. *Arch Gen Psychiatry, 54*(2), 159-165. <https://doi.org/10.1001/archpsyc.1997.01830140071013>

Hagler, D. J., Jr., Hatton, S., Cornejo, M. D., Makowski, C., Fair, D. A., Dick, A. S., Sutherland, M. T., Casey, B. J., Barch, D. M., Harms, M. P., Watts, R., Bjork, J. M., Garavan, H. P., Hilmer, L., Pung, C. J., Sicat, C. S., Kuperman, J., Bartsch, H., Xue, F., Heitzeg, M. M., Laird, A. R., Trinh, T. T., Gonzalez, R., Tapert, S. F., Riedel, M. C., Squeglia, L. M., Hyde, L. W., Rosenberg, M. D., Earl, E. A., Howlett, K. D., Baker, F. C., Soules, M., Diaz, J., de Leon, O. R., Thompson, W. K., Neale, M. C., Herting, M., Sowell, E. R., Alvarez, R. P., Hawes, S. W., Sanchez, M., Bodurka, J., Breslin, F. J., Morris, A. S., Paulus, M. P., Simmons, W. K., Polimeni, J. R., van der Kouwe, A., Nencka, A. S., Gray, K. M., Pierpaoli, C., Matochik, J. A., Noronha, A., Aklin, W. M., Conway, K., Glantz, M., Hoffman, E., Little, R., Lopez, M., Pariyadath, V., Weiss, S. R., Wolff-Hughes, D. L., DelCarmen-Wiggins, R., Feldstein Ewing, S. W., Miranda-Dominguez, O., Nagel, B. J., Perrone, A. J., Sturgeon, D. T., Goldstone, A., Pfefferbaum, A., Pohl, K. M., Prouty, D., Uban, K., Bookheimer, S. Y., Dapretto, M., Galvan, A., Bagot, K., Giedd, J., Infante, M. A., Jacobus, J., Patrick, K., Shilling, P. D., Desikan, R., Li, Y., Sugrue, L., Banich, M. T., Friedman, N., Hewitt, J. K., Hopfer, C., Sakai, J., Tanabe, J., Cottler, L. B., Nixon, S. J., Chang, L., Cloak, C., Ernst, T., Reeves, G., Kennedy, D. N., Heeringa, S., Peltier, S., Schulenberg, J., Sripada, C., Zucker, R. A., Iacono, W. G., Luciana, M., Calabro, F. J., Clark, D. B., Lewis, D. A., Luna, B., Schirda, C., Brima, T., Foxe, J. J., Freedman, E. G., Mruzek, D. W., Mason, M. J., Huber, R., McGlade, E., Prescot, A., Renshaw, P. F., Yurgelun-Todd, D. A., Allgaier, N. A., Dumas, J. A., Ivanova, M., Potter, A., Florsheim, P., Larson, C., Lisdahl, K., Charness, M. E., Fuemmeler, B., Hettema, J. M., Maes, H. H., Steinberg, J., Anokhin, A. P., Glaser, P., Heath, A. C., Madden, P. A., Baskin-Sommers, A., Constable, R. T., Grant, S. J., Dowling, G. J., Brown, S. A., Jernigan, T. L., & Dale, A. M. (2019, Nov 15). Image processing and analysis methods for the Adolescent Brain Cognitive Development Study. *Neuroimage, 202*, 116091. <https://doi.org/10.1016/j.neuroimage.2019.116091>

Hodes, R. J., Insel, T. R., Landis, S. C., & Research, N. I. H. B. f. N. (2013, Mar 12). The NIH toolbox: setting a standard for biomedical research. *Neurology, 80*(11 Suppl 3), S1. <https://doi.org/10.1212/WNL.0b013e3182872e90>

Huppertz, C., Bartels, M., de Zeeuw, E. L., van Beijsterveldt, C. E. M., Hudziak, J. J., Willemsen, G., Boomsma, D. I., & de Geus, E. J. C. (2016, Sep). Individual Differences in Exercise Behavior: Stability and Change in Genetic and Environmental Determinants From Age 7 to 18. *Behav Genet, 46*(5), 665-679. <https://doi.org/10.1007/s10519-016-9799-x>

Luciana, M., Bjork, J. M., Nagel, B. J., Barch, D. M., Gonzalez, R., Nixon, S. J., & Banich, M. T. (2018, Aug). Adolescent neurocognitive development and impacts of substance use: Overview of the adolescent brain cognitive development (ABCD) baseline neurocognition battery. *Dev Cogn Neurosci, 32*, 67-79. <https://doi.org/10.1016/j.dcn.2018.02.006>

Rueda, M. R., Fan, J., McCandliss, B. D., Halparin, J. D., Gruber, D. B., Lercari, L. P., & Posner, M. I. (2004). Development of attentional networks in childhood. *Neuropsychologia, 42*(8), 1029-1040. <https://doi.org/10.1016/j.neuropsychologia.2003.12.012>

Salthouse, T. A., Babcock, R. L., & Shaw, R. J. (1991, Mar). Effects of adult age on structural and operational capacities in working memory. *Psychol Aging, 6*(1), 118-127. <https://doi.org/10.1037//0882-7974.6.1.118>

Sharif, I., Wills, T. A., & Sargent, J. D. (2010, Jan). Effect of visual media use on school performance: a prospective study. *J Adolesc Health, 46*(1), 52-61. <https://doi.org/10.1016/j.jadohealth.2009.05.012>

Thompson, W. K., Barch, D. M., Bjork, J. M., Gonzalez, R., Nagel, B. J., Nixon, S. J., & Luciana, M. (2019, Apr). The structure of cognition in 9 and 10 year-old children and associations with problem behaviors: Findings from the ABCD study's baseline neurocognitive battery. *Dev Cogn Neurosci, 36*, 100606. <https://doi.org/10.1016/j.dcn.2018.12.004>

Tulsky, D. S., Carlozzi, N., Chiaravalloti, N. D., Beaumont, J. L., Kisala, P. A., Mungas, D., Conway, K., & Gershon, R. (2014, Jul). NIH Toolbox Cognition Battery (NIHTB-CB): list sorting test to measure working memory. *J Int Neuropsychol Soc, 20*(6), 599-610. <https://doi.org/10.1017/S135561771400040X>

Tulsky, D. S., Carlozzi, N. E., Chevalier, N., Espy, K. A., Beaumont, J. L., & Mungas, D. (2013, Aug). V. NIH Toolbox Cognition Battery (CB): measuring working memory. *Monogr Soc Res Child Dev, 78*(4), 70-87. <https://doi.org/10.1111/mono.12035>

Wechsler, D. (2014). *Wechsler intelligence scale for children®–fifth edition.* Pearson.

Zelazo, P. D. (2006). The Dimensional Change Card Sort (DCCS): a method of assessing executive function in children. *Nat Protoc, 1*(1), 297-301. <https://doi.org/10.1038/nprot.2006.46>

Zelazo, P. D., Anderson, J. E., Richler, J., Wallner-Allen, K., Beaumont, J. L., Conway, K. P., Gershon, R., & Weintraub, S. (2014, Jul). NIH Toolbox Cognition Battery (CB): validation of executive function measures in adults. *J Int Neuropsychol Soc, 20*(6), 620-629. <https://doi.org/10.1017/S1355617714000472>

Zelazo, P. D., Anderson, J. E., Richler, J., Wallner-Allen, K., Beaumont, J. L., & Weintraub, S. (2013, Aug). II. NIH Toolbox Cognition Battery (CB): measuring executive function and attention. *Monogr Soc Res Child Dev, 78*(4), 16-33. <https://doi.org/10.1111/mono.12032>

## Prior Genetically-Informative Studies

### 1. Structural Neuroimaging

The brain imaging results described here are likely to be useful to researchers in general as the number of MZ and DZ twin pairs in the ABCD Study exceeds those of all prior MRI studies of youth. The ABCD Study provides information regarding brain measures of morphometry with measures for both grey and white matter, as well as information on brain function, both at rest and during task performance. One motive for understanding which aspects of brain structure and function are heritable is that these neural features are potential endophenotypes that mediate between gene expression and behavioral, psychological and clinical outcomes (Iacono et al., 2017; Kendler & Neale, 2010). Here, we discuss the heritability of structural brain features, focusing on regions most relevant to adolescent development: subcortical structures involved in reward and emotion, and prefrontal regions involved in cognitive control. Measures of brain structure fall into two broad categories. The first examines the structure of specific brain regions, at different levels of spatial resolution from whole brain to lobes, specific gyri, architectonic divisions (e.g., brain regions) or vertices. A primary focus is on the characteristics of grey matter (i.e., neuronal bodies). The second category examines characteristics of white matter that serve as the connective filaments that link distal brain regions.

#### 1.1. Regional Measures of Morphometry

Prior genetically-informative neuroimaging studies (Blokland et al., 2012; Peper et al., 2007) have primarily investigated endophenotypes derived from structural magnetic resonance imaging (sMRI). Although the acquisition of sMRI data is technically complicated, the resultant volumetric images can be conceptualized as down-sampled, digital models of the anatomy of the human brain. Raw sMRI data can be postprocessed to obtain quantitative measures at multiple levels of spatial resolution, from global to measures as small as a few millimeters in size. Neuroanatomical regions of interest (ROIs) are intermediate to these two extremes, and typically conform to gyrus-level anatomy or cytoarchitectural divisions. In addition to total (gray + white matter) volumes, sMRI images contain information on other neuroanatomic traits that can be extracted via additional postprocessing steps; the most common measures include cortical thickness, cortical surface area, gyrification index, gray matter density, and separately, gray and white matter volumes. Cortical thickness, typically defined as the distance from white matter to the pial surface, and cortical surface area, appear to be driven by distinct genetic mechanisms (Panizzon et al., 2009) and are typically examined separately. Their product is highly related to, but not exactly equivalent to, cortical volume. Similar to total volumes, these other measures can be examined at multiple levels of spatial resolution.

The extant twin literature has consistently found that phenotypic variation is primarily associated with additive genetic sources for most **global brain measures** in most populations. For example, a meta-analysis (including both children and adults) estimated heritabilities of 83% for total brain volume, 72% for total gray matter volume, and 85% for total white matter volume (Blokland et al., 2012). Heritability estimates for most **cerebral lobar and subcortical** (e.g., thalamic) **volumes** are moderate to high, with most regions having heritability estimates greater than 50% (Christova et al., 2021). The literature on **non-volumetric measures** is sparser, but the heritabilities of total cerebral surface area and cortical thickness in adults have been estimated as high as 95% and 81%, respectively (Eyler et al., 2011; Panizzon et al., 2009). Gyral-level measures of surface area heritability are also very high (>75%) in adults, while cortical thickness is somewhat lower and more regionally variable (Blokland et al., 2012; Eyler et al., 2012). It is noteworthy that smaller ROIs are more likely to have lower heritability estimates, a finding that may be partly due to increased measurement error. When neuroanatomy is measured at **ROI or voxel-level** **scales**, regional patterns emerge. For example, in a landmark study, the strongest evidence of genetic effects on gray matter density were in dorsal frontal lobes and language centers (Thompson et al., 2001), which is consistent with the high heritability of cognitive abilities (Plomin & Deary, 2015). Similar patterns have been observed for cortical thickness in children (Lenroot et al., 2009).

The rapid **neurodevelopmental changes** observed during childhood present particular challenges to behavior genetic analysis, but also make this an important population for exploring gene-brain relationships (Dima et al., 2022; Jansen et al., 2015). Strong genetic influences on brain structure can be observed even during the neonatal period; similar to adults, high heritabilities have been observed for both global volumes and total cerebral surface area by ~40 weeks of gestation (Gilmore et al., 2010; Jha et al., 2018). High heritabilities for most large ROIs are also observed in older children and adolescents, with strong genetic influences on global and lobar volumes and cerebral surface area (Peper et al., 2009; Wallace et al., 2006). Despite many similarities to the adult population, there are also important differences. For example, in neonatal, childhood, and emerging adult samples (Jha et al., 2018; Schmitt, Raznahan, et al., 2019; Strike et al., 2019; Yoon et al., 2012) heritability for surface area in posterior brain regions along the midline is very high (*h^2^*=~90%), whereas in adults it is smaller (*h^2^*=~60%) (Eyler et al., 2012; McKay et al., 2015). Additionally, estimates of average cerebral cortical thickness are substantially lower in neonates (*h^2^*=29%) and children (*h^2^*=44%) compared to adults (Jha et al., 2018; Schmitt, Neale, et al., 2019). These observations suggest that the heritability of cortical thickness is dynamic, a hypothesis supported by the few genetically-informative longitudinal studies on neuroimaging phenotypes in children (Schmitt et al., 2014; Teeuw et al., 2019). The precise etiology for dynamic changes in heritability of cortical thickness is unclear, and remains an area of active investigation. In contrast, there is evidence that additive genetic effects on subcortical structures are relatively stable in late childhood and adolescence (Swagerman et al., 2014). Unlike in older adults, where studies have suggested that surface area and cortical thickness are genetically orthogonal, significant genetic correlations between these measures have been observed in childhood (Jha et al., 2018; Schmitt, Neale, et al., 2019). Thus, the characterization of genetic influences on the brain requires neurodevelopmental context.

#### 1.2. Anatomical Measures of Connectivity

Another interesting set of structural features in the human brain concerns white matter, which consists of myelinated axons of neurons that allow for connections between brain regions. The method used to extract such information, diffusion imaging (dMRI), provides information on how water diffuses in neural tissue, and examines the directionality of such diffusion as a proxy for white matter integrity and directionality so as to reconstruct the architecture of white matter tracts (Mori & Zhang, 2006). One typically-investigated measure is **fractional anisotropy** (FA), the degree to which water diffuses in a specific direction, which is high in white matter tracts and lower in cerebrospinal fluid, and is thought to be a maker of normal brain development reflecting myelination, axonal diameter and fiber density. FA can be further parcellated into radial and axial diffusivity, the extent to which water diffuses perpendicular to, and in parallel with, fiber tracts. Another metric is **mean diffusivity** (MD), which describes the average magnitude of water diffusion in all directions within brain tissue. While relatively non-specific, high values of MD are thought to reflect decreased white matter integrity (Alexander et al., 2007; Jutten et al., 2019; Soares et al., 2013).

In adults, there appears to be a general genetic influence on measures across all major white matter tracts accounting for ~50% of variation in FA and 62% in MD (Gustavson et al., 2019). Above and beyond this general genetic influence common to all tracts, there are also genetic influences that vary across tracts and on average explain another ~10-20% of the variance, such that heritability of white matter tract measures appears to be on the order of 66-75% (Gustavson et al., 2019). Strikingly similar estimates of mean heritability are found when examining small children (Lee et al., 2019). However, other adult twin studies using the Human Connectome Project (HCP) data suggest higher values for heritability of 11 major white matter tracts (*h^2^*=66-90%, average FA 88%) (Best et al., 2020; Christova et al., 2021; Kochunov et al., 2015). In contrast, heritability estimates from two dMRI datasets from the ENIGMA project in adult samples suggest somewhat lower heritability of white matter (*h^2^*=~66-75%), as did a study of emerging adults (*h^2^*=40%) but not adolescents (*h^2^*=70-80%) (Chiang et al., 2011). Further, heritability estimates of inter-regional structural connectivities (dMRI streamlines, *h^2^*=~25-80%) from the HCP twin sample (Zhang et al., 2018) suggest a greater proportion of genetic variance in functional connectivity between nodes connected by major white matter tracts, such as the arcuate fasciculus, uncinate fasciculus, optic radiation, and portions of corpus callosum (CC). Despite some variation, the picture that emerges from prior studies is one of high heritabilities of white matter metrics that, for the most part, vary little from childhood to adulthood.

### 2. Neurocognition

Phenotypic variance in higher-order cognition that can be attributed to genetic versus environmental mechanisms has been extensively examined using twin and adoption methods (Bouchard & McGue, 1981; Polderman et al., 2015). Cognition can be parsed into general ability (*g*) and discrete abilities such as attention, perceptual skills, learning, memory, and processing speed that are distinct from *g* but contribute to it. General ability can also be sub-divided into two domains. The first is fluid reasoning, the ability to solve novel problems and engage in new learning, which is thought to be biologically based and culture free. The second, crystallized reasoning, is based on acquired knowledge, is more culturally-influenced, and associated with educational attainment (Cattell, 1963).

In light of the high heritability values detected in brain morphometric structures that govern cognition, it seems likely that heritability of cognitive abilities would roughly follow suit. Indeed, **general cognitive ability** (*g*) and its facets, typically measured by intelligence quotient (IQ), which is derived from scores across a battery of fluid and crystallized measures, or through factor analytic techniques that quantify the proportion of variance that is common to a group of measured tasks, show moderate to high heritabilities in adults (Neisser et al., 1996; Plomin & DeFries, 1980). Estimates have remained relatively stable across samples using a variety of quantitative methods. For instance, an early examination of twin correlations in general ability, derived from 19 studies, reported a median MZ correlation (rMZ) of 0.86 and DZ correlation (rDZ) of 0.62 (Loehlin & Nichols, 1976), consistent with a heritability ~48%. A subsequent report that aggregated data across 113,942 familial pairings, including twins reared together or apart, as well as sibling, parent-offspring pairs and other familial pairings, largely based on individuals under the age of 20, reported heritability estimates of 50% (Bouchard & McGue, 1981; McGue et al., 1993). Other family-based studies have since affirmed, with increasingly sophisticated quantitative methods, that the heritability of general cognitive ability, including both fluid and crystallized domains, is in the range of 50-70% in adults (Briley & Tucker-Drob, 2013; Deary et al., 2009; van Soelen et al., 2011; Wadsworth et al., 2014), though its genetic architecture may be a complex blend of non-additive genetic and environmental sources (Fulker & Eysenck, 1979).

Importantly, there are **developmental trends** in heritability estimates and in the nature of environmental influences. Intraclass correlations and SEM methodologies indicate that heritabilities increase markedly from childhood into adulthood for *g*  (Brant et al., 2009; Haworth et al., 2010; McGue et al., 1993). In children, the influence of shared environment can be substantial, accounting for 10-33% of the variance in general ability, but this source of variation declines markedly by adolescence and approaches zero by adulthood (Wadsworth et al., 2014). The remaining variance, due to non-shared environment, increases through childhood reaching high levels in adulthood (Friedman et al., 2008; Haworth et al., 2010). Some studies have considered whether heritabilities are impacted by demographic or premorbid characteristics. There is little support for differential sources of phenotypic variation by sex or by individual differences in levels of ability (Wadsworth et al., 2014).

In contrast to what has been reported for *g,* heritability estimates for **specific abilities** are more moderate and variable, depending on the particular task. For instance, a recent meta-analysis of 37 twin studies (N=41,623 pairs) focused on spatial ability (King et al., 2019) found high heritabilities (average *h^2^*=61%) with moderate contributions from non-shared (average *e^2^*=43%) and shared environment (average *c^2^*=7%). Effects did not vary by sex or by type of spatial ability examined. However, children and younger adolescents had estimates of shared environmental contributions significantly greater than zero (average *c^2^*=15%), while other developmental groups did not.

Individual **executive function** (EF) domains, reflected by performance on laboratory measures of inhibitory control, working memory/updating and cognitive flexibility have been examined, in both adolescents and adults, with conflicting findings. Several studies cohere in suggesting moderate genetic and unshared environmental influences on specific EF measures (Anokhin et al., 2011) with negligible contributions from shared environment (Ando et al., 2001; Anokhin et al., 2004; Fan et al., 2001; Malone & Iacono, 2002; Posthuma et al., 2002; Stins et al., 2004). However, using a latent variable approach on 9 EF tasks and a full Wechsler battery (WAIS-III) based on a conceptual model of EF found low to moderate levels of genetic variation in EF abilities (Friedman et al., 2008; Miyake et al., 2000). The one analysis to date of heritability of specific NIH Toolbox tasks, which used Human Connectome Project data (Pinto et al., 2020), indicated heritabilities of 49% for general EF and 37% for picture sequence memory. Variation in a common EF factor, derived from a hierarchical ACE model and distinct from both IQ and perceptual speed, was entirely attributable to genetic variation (Friedman et al., 2008), a finding which was replicated in young twins using a different task battery (Engelhardt et al., 2015). Independent genetic influences were found for updating and shifting components of EF but not inhibition, suggesting executive functions may be influenced differently by genetic versus environmental sources of variation. In sum, with respect to EF, genetic factors that distinguish specific abilities from one another may express themselves differently in childhood versus adulthood with more modest heritability, whereas the latent variable that accounts for most of the variance in performance across a variety of EF tasks itself appears to be highly heritable from middle childhood onward.

Sociodemographic characteristics may impact heritability estimates. In the United States and countries with limited economic entitlement programs such as socialized medicine, estimates vary as a function of ability level (Haworth et al., 2009) and SES (Turkheimer & Horn, 2014) such that heritability for cognition appears lower in lower SES groups, a pattern that could reflect differences in measurement error between groups or greater impacts of the environment at extremes of the SES distribution, or both.

In sum, heritabilities for general cognitive abilities increase markedly from childhood to young adulthood, accounting for roughly 50% of phenotypic variance. This pattern leaves room for significant environmental influence, most of which appears unshared between twins. The influence of shared environment is substantial in early childhood but decreases into adolescence. As the structure of cognition changes over time from middle childhood into young adulthood (Thompson et al., 2019), the ABCD sample affords an unprecedented opportunity to assess these developmental patterns and how genetic influences on behavior map onto brain-based changes.

### 3. Child Psychopathology

The 112-item Child Behavior Checklist (CBCL: (Achenbach & Edelbrock, 1981, 1983) is a widely-used instrument for parents to rate their children’s **externalizing** and **internalizing** **behavior.** It also yields subscale scores. ABCD also collects teacher ratings on an 18-item Brief Problem Monitor (BPM) scored for attention problems, and Internalizing and Externalizing behaviors (Achenbach & Edelbrock, 1981). The CBCL and its derived scores have been widely used in behavior genetic research, especially twin studies. Individual differences in both internalizing and externalizing behaviors appear moderately heritable. Shared environment is smaller, relative to genetic influences, for developmentally stable problem behaviors and for behavior that is consistent across more than one setting (e.g., home and school) or rated by more than one rater (Haberstick et al., 2005, 2006). While there is considerable stability of problem behavior, there are also changes during development, and there may be differences between boys and girls in the causes of individual differences (van der Valk et al., 2003).

However, these broad characterizations are complicated by the nature of the CBCL assessment, which relies on **parental reports**. This complication is especially important in the ABCD Study^®^ where only one parent is reporting, typically the mother. As Neale and Stevenson (Neale & Stevenson, 1989) and Hewitt et al (Hewitt et al., 1992) have noted, parental ratings reflect both the characteristics of the child being rated *and* the characteristics of the parent as rater. When only one parent rates both of their twins, rater bias potentially manifests itself as a higher twin correlation. If rater bias applies to twin pairs irrespective of zygosity and it increases both MZ and DZ correlations, the result will be an increase in the apparent influence of the shared or family environment. In other circumstances, a rater may *contrast* the members of a twin pair, increasing the within family variance and potentially decreasing the twin pair correlations. If different raters rate each twin, e.g., different teachers or different parents, then rater biases or different perspectives will inflate the estimate of E, the within family environmental effect (Bartels et al., 2004; Haberstick et al., 2005). Considering studies where both parents provided ratings of young twins, Hewitt et al (1992) found that more than half of the shared environmental influence estimated in maternal ratings can be attributed to rater bias, and most of the unique environmental influence to unreliability or inconsistency between ratings. The heritability of internalizing behaviors in young boys rated consistently by both parents may be as high as 70%.

As we consider the results reported here for the initial univariate results for the CBCL assessments of externalizing and internalizing behaviors and subscales, our interpretation of A, C, and E components of variance requires context. Of particular note are: situational specificity (e.g., home versus school), generality, stability and change in behavior across development, and, importantly, the source of these assessments (a parent in the case of the CBCL, a teacher in the case of the BPM). Developmental and multivariate analyses will provide insights into these issues.

### 4. Physical and Other Traits

**Height and weight** have been extensively researched in twin studies; both are in the top 10 most-studied traits in twin studies (Polderman et al., 2015). Height is relatively easy to measure accurately, although some self-report biases are known (Perez et al., 2015). Results consistently find rMZs to be around 0.91 and rDZs=0.54, which yield variance component estimates of 73% for additive genetic factors and 18% for shared environment. The correlations are reasonably consistent across age; at 0-11 years MZ and DZ correlations are 0.88 and 0.59 respectively, while for 12-17 they are 0.94 and 0.49, suggesting high to very high heritability for height (Polderman et al., 2015). Estimates of genetic variance of height across large European cohorts (Dubois et al., 2012; Silventoinen et al., 2007) were generally higher than for East Asians (Hur et al., 2008; Liu et al., 2015), with larger shared environmental contributions in North America/Australia (Jelenkovic et al., 2016). Twin study results for weight are similar for childhood ages, but estimates of heritability tend to be slightly less than for height (Dubois et al., 2012; Hur et al., 2008; Liu et al., 2015). Much prior genetic research has focused on **body mass index** (BMI, measured as weight in kilograms divided by the square of height in meters), because it is easily measured and associates with adverse health outcomes. Genetic factors account for the majority of variance in BMI (*h^2^*=50-90%), even in early adolescence (Haworth et al., 2008; Nan et al., 2012; Silventoinen et al., 2017), with estimates from twin studies typically higher than from family studies (Elks et al., 2012; Maes et al., 1997). A meta-analysis suggested that heritability of BMI was high in children and increased up to age 18 (Elks et al., 2012). These trends were confirmed in two large mega-analyses showing *h^2^* increasing from 55% in children to ~75% in young adults (Silventoinen et al., 2016). Shared environment factors were significant prior to puberty and highest at ages 4-7.

Several studies have investigated the heritability of **hormone levels** in adolescence. For DHEA, additive genetic factors accounted for 50-60% in boys and 10-65% in girls (Grotzinger, Briley, et al., 2018; Hoekstra et al., 2006; Li et al., 2017; Van Hulle et al., 2015), with moderate shared environmental influences. Estimates for testosterone differed between boys and girls, with higher heritabilities for boys and higher shared environmental contributions for girls. Familial resemblances (A+C) were consistently above 50% of the variance for hormone level measures (Grotzinger, Briley, et al., 2018; Grotzinger, Mann, et al., 2018; Harden et al., 2014; Harris et al., 1998; Koenis et al., 2013; Van Hulle et al., 2015). Literature on the heritability of **sleep** characteristics in adolescence is limited, with moderate to high estimates for sleep efficiency (*h^2^*=32-52%) and sleep latency (*h^2^*=72-83%) and mixed results for sleep time (Rusterholz et al., 2018; Sletten et al., 2013) in early adolescence. Shared environmental factors explained most of the variance for sleep measures during school days, whereas genetic factors accounted for most of the variance on free days (Inderkum & Tarokh, 2018). We are aware of no previous studies of adolescent twins on **screen time**, **listening to music** and **reading for pleasure**, although online media use has been explored (Ayorech et al., 2017) suggesting substantial heritability. An expansive set of measures of home and environmental conditions, including SES, were assessed. A large proportion of shared environmental variation is expected for these measures, as they are unlikely to differ between members of a twin pair, regardless of their zygosity.

In sum, the aims of this paper are to generate univariate estimates of heritability and environmental sources of variance for baseline brain and behavioral measures in twins ascertained at birth assessed at ages 9-10; assessing the validity of both the twin data and methodological approach, and to provide easy access to these results for thousands of variables through a web interface as a starting point for future in-depth applications. This paper will focus on continuous variables assessing structural neuroimaging, neurocognition, childhood psychopathology, physical and other traits. Companion papers will detail results for functional neuroimaging and for binary and ordinal variables.

### References

Achenbach, T. M., & Edelbrock, C. S. (1981). Behavioral problems and competencies re­ ported by parents of normal and disturbed children aged four through sixteen. *Monogr. Soc. Res. Child Dev., 46*.

Achenbach, T. M., & Edelbrock, C. S. (1983). *Manual for the Child Behavior Checklist and Revised Child Behavior Profile.* Dep. Psychiatry, Univ. Vermont.

Alexander, A. L., Lee, J. E., Lazar, M., & Field, A. S. (2007, Jul). Diffusion tensor imaging of the brain. *Neurotherapeutics, 4*(3), 316-329. <https://doi.org/10.1016/j.nurt.2007.05.011>

Ando, J., Ono, Y., & Wright, M. J. (2001, Nov). Genetic structure of spatial and verbal working memory. *Behav Genet, 31*(6), 615-624. <https://doi.org/10.1023/a:1013353613591>

Anokhin, A. P., Golosheykin, S., Grant, J. D., & Heath, A. C. (2011, Mar). Heritability of delay discounting in adolescence: a longitudinal twin study. *Behav Genet, 41*(2), 175-183. <https://doi.org/10.1007/s10519-010-9384-7>

Anokhin, A. P., Heath, A. C., & Myers, E. (2004, Sep 30). Genetics, prefrontal cortex, and cognitive control: a twin study of event-related brain potentials in a response inhibition task. *Neurosci Lett, 368*(3), 314-318. <https://doi.org/10.1016/j.neulet.2004.07.036>

Ayorech, Z., von Stumm, S., Haworth, C. M., Davis, O. S., & Plomin, R. (2017). Personalized Media: A Genetically Informative Investigation of Individual Differences in Online Media Use. *PLoS One, 12*(1), e0168895. <https://doi.org/10.1371/journal.pone.0168895>

Bartels, M., Boomsma, D. I., Hudziak, J. J., Rietveld, M. J., van Beijsterveldt, T. C., & van den Oord, E. J. (2004, Apr). Disentangling genetic, environmental, and rater effects on internalizing and externalizing problem behavior in 10-year-old twins. *Twin Res, 7*(2), 162-175. <https://doi.org/10.1375/136905204323016140>

Best, J. R., Dao, E., Churchill, R., & Cosco, T. D. (2020). Associations Between Physical Fitness and Brain Structure in Young Adulthood. *Front Psychol, 11*, 608049. <https://doi.org/10.3389/fpsyg.2020.608049>

Blokland, G. A., de Zubicaray, G. I., McMahon, K. L., & Wright, M. J. (2012, Jun). Genetic and environmental influences on neuroimaging phenotypes: a meta-analytical perspective on twin imaging studies. *Twin Res Hum Genet, 15*(3), 351-371. <https://doi.org/10.1017/thg.2012.11>

Bouchard, T. J., Jr., & McGue, M. (1981, May 29). Familial studies of intelligence: a review. *Science, 212*(4498), 1055-1059. <https://doi.org/10.1126/science.7195071>

Brant, A. M., Haberstick, B. C., Corley, R. P., Wadsworth, S. J., DeFries, J. C., & Hewitt, J. K. (2009, Jul). The developmental etiology of high IQ. *Behav Genet, 39*(4), 393-405. <https://doi.org/10.1007/s10519-009-9268-x>

Briley, D. A., & Tucker-Drob, E. M. (2013, Sep). Explaining the increasing heritability of cognitive ability across development: a meta-analysis of longitudinal twin and adoption studies. *Psychol Sci, 24*(9), 1704-1713. <https://doi.org/10.1177/0956797613478618>

Cattell, R. B. (1963). Theory of fluid and crystallized intelligence: A critical experiment. *Journal of Educational Psychology, 54*, 1–22.

Chiang, M. C., McMahon, K. L., de Zubicaray, G. I., Martin, N. G., Hickie, I., Toga, A. W., Wright, M. J., & Thompson, P. M. (2011, Feb 1). Genetics of white matter development: a DTI study of 705 twins and their siblings aged 12 to 29. *Neuroimage, 54*(3), 2308-2317. <https://doi.org/10.1016/j.neuroimage.2010.10.015>

Christova, P., Joseph, J., & Georgopoulos, A. P. (2021, Apr). Human Connectome Project: heritability of brain volumes in young healthy adults. *Exp Brain Res, 239*(4), 1273-1286. <https://doi.org/10.1007/s00221-021-06057-0>

Deary, I. J., Johnson, W., & Houlihan, L. M. (2009, Jul). Genetic foundations of human intelligence. *Hum Genet, 126*(1), 215-232. <https://doi.org/10.1007/s00439-009-0655-4>

Dima, D., Modabbernia, A., Papachristou, E., Doucet, G. E., Agartz, I., Aghajani, M., Akudjedu, T. N., Albajes-Eizagirre, A., Alnaes, D., Alpert, K. I., Andersson, M., Andreasen, N. C., Andreassen, O. A., Asherson, P., Banaschewski, T., Bargallo, N., Baumeister, S., Baur-Streubel, R., Bertolino, A., Bonvino, A., Boomsma, D. I., Borgwardt, S., Bourque, J., Brandeis, D., Breier, A., Brodaty, H., Brouwer, R. M., Buitelaar, J. K., Busatto, G. F., Buckner, R. L., Calhoun, V., Canales-Rodriguez, E. J., Cannon, D. M., Caseras, X., Castellanos, F. X., Cervenka, S., Chaim-Avancini, T. M., Ching, C. R. K., Chubar, V., Clark, V. P., Conrod, P., Conzelmann, A., Crespo-Facorro, B., Crivello, F., Crone, E. A., Dannlowski, U., Dale, A. M., Davey, C., de Geus, E. J. C., de Haan, L., de Zubicaray, G. I., den Braber, A., Dickie, E. W., Di Giorgio, A., Doan, N. T., Dorum, E. S., Ehrlich, S., Erk, S., Espeseth, T., Fatouros-Bergman, H., Fisher, S. E., Fouche, J. P., Franke, B., Frodl, T., Fuentes-Claramonte, P., Glahn, D. C., Gotlib, I. H., Grabe, H. J., Grimm, O., Groenewold, N. A., Grotegerd, D., Gruber, O., Gruner, P., Gur, R. E., Gur, R. C., Hahn, T., Harrison, B. J., Hartman, C. A., Hatton, S. N., Heinz, A., Heslenfeld, D. J., Hibar, D. P., Hickie, I. B., Ho, B. C., Hoekstra, P. J., Hohmann, S., Holmes, A. J., Hoogman, M., Hosten, N., Howells, F. M., Hulshoff Pol, H. E., Huyser, C., Jahanshad, N., James, A., Jernigan, T. L., Jiang, J., Jonsson, E. G., Joska, J. A., Kahn, R., Kalnin, A., Kanai, R., Klein, M., Klyushnik, T. P., Koenders, L., Koops, S., Kramer, B., Kuntsi, J., Lagopoulos, J., Lazaro, L., Lebedeva, I., Lee, W. H., Lesch, K. P., Lochner, C., Machielsen, M. W. J., Maingault, S., Martin, N. G., Martinez-Zalacain, I., Mataix-Cols, D., Mazoyer, B., McDonald, C., McDonald, B. C., McIntosh, A. M., McMahon, K. L., McPhilemy, G., Meinert, S., Menchon, J. M., Medland, S. E., Meyer-Lindenberg, A., Naaijen, J., Najt, P., Nakao, T., Nordvik, J. E., Nyberg, L., Oosterlaan, J., de la Foz, V. O., Paloyelis, Y., Pauli, P., Pergola, G., Pomarol-Clotet, E., Portella, M. J., Potkin, S. G., Radua, J., Reif, A., Rinker, D. A., Roffman, J. L., Rosa, P. G. P., Sacchet, M. D., Sachdev, P. S., Salvador, R., Sanchez-Juan, P., Sarro, S., Satterthwaite, T. D., Saykin, A. J., Serpa, M. H., Schmaal, L., Schnell, K., Schumann, G., Sim, K., Smoller, J. W., Sommer, I., Soriano-Mas, C., Stein, D. J., Strike, L. T., Swagerman, S. C., Tamnes, C. K., Temmingh, H. S., Thomopoulos, S. I., Tomyshev, A. S., Tordesillas-Gutierrez, D., Trollor, J. N., Turner, J. A., Uhlmann, A., van den Heuvel, O. A., van den Meer, D., van der Wee, N. J. A., van Haren, N. E. M., Van't Ent, D., van Erp, T. G. M., Veer, I. M., Veltman, D. J., Voineskos, A., Volzke, H., Walter, H., Walton, E., Wang, L., Wang, Y., Wassink, T. H., Weber, B., Wen, W., West, J. D., Westlye, L. T., Whalley, H., Wierenga, L. M., Williams, S. C. R., Wittfeld, K., Wolf, D. H., Worker, A., Wright, M. J., Yang, K., Yoncheva, Y., Zanetti, M. V., Ziegler, G. C., Thompson, P. M., Frangou, S., & Karolinska Schizophrenia, P. (2022, Jan). Subcortical volumes across the lifespan: Data from 18,605 healthy individuals aged 3-90 years. *Hum Brain Mapp, 43*(1), 452-469. <https://doi.org/10.1002/hbm.25320>

Dubois, L., Ohm Kyvik, K., Girard, M., Tatone-Tokuda, F., Perusse, D., Hjelmborg, J., Skytthe, A., Rasmussen, F., Wright, M. J., Lichtenstein, P., & Martin, N. G. (2012). Genetic and environmental contributions to weight, height, and BMI from birth to 19 years of age: an international study of over 12,000 twin pairs. *PLoS One, 7*(2), e30153. <https://doi.org/10.1371/journal.pone.0030153>

Elks, C. E., den Hoed, M., Zhao, J. H., Sharp, S. J., Wareham, N. J., Loos, R. J., & Ong, K. K. (2012). Variability in the heritability of body mass index: a systematic review and meta-regression. *Front Endocrinol (Lausanne), 3*, 29. <https://doi.org/10.3389/fendo.2012.00029>

Engelhardt, L. E., Briley, D. A., Mann, F. D., Harden, K. P., & Tucker-Drob, E. M. (2015, Aug). Genes Unite Executive Functions in Childhood. *Psychol Sci, 26*(8), 1151-1163. <https://doi.org/10.1177/0956797615577209>

Eyler, L. T., Chen, C. H., Panizzon, M. S., Fennema-Notestine, C., Neale, M. C., Jak, A., Jernigan, T. L., Fischl, B., Franz, C. E., Lyons, M. J., Grant, M., Prom-Wormley, E., Seidman, L. J., Tsuang, M. T., Fiecas, M. J., Dale, A. M., & Kremen, W. S. (2012, Jun). A comparison of heritability maps of cortical surface area and thickness and the influence of adjustment for whole brain measures: a magnetic resonance imaging twin study. *Twin Res Hum Genet, 15*(3), 304-314. <https://doi.org/10.1017/thg.2012.3>

Eyler, L. T., Prom-Wormley, E., Panizzon, M. S., Kaup, A. R., Fennema-Notestine, C., Neale, M. C., Jernigan, T. L., Fischl, B., Franz, C. E., Lyons, M. J., Grant, M., Stevens, A., Pacheco, J., Perry, M. E., Schmitt, J. E., Seidman, L. J., Thermenos, H. W., Tsuang, M. T., Chen, C. H., Thompson, W. K., Jak, A., Dale, A. M., & Kremen, W. S. (2011, Oct). Genetic and environmental contributions to regional cortical surface area in humans: a magnetic resonance imaging twin study. *Cereb Cortex, 21*(10), 2313-2321. <https://doi.org/10.1093/cercor/bhr013>

Fan, J., Wu, Y., Fossella, J. A., & Posner, M. I. (2001). Assessing the heritability of attentional networks. *BMC Neurosci, 2*, 14. <https://doi.org/10.1186/1471-2202-2-14>

Friedman, N. P., Miyake, A., Young, S. E., DeFries, J. C., Corley, R. P., & Hewitt, J. K. (2008, May). Individual differences in executive functions are almost entirely genetic in origin. *J Exp Psychol Gen, 137*(2), 201-225. <https://doi.org/10.1037/0096-3445.137.2.201>

Fulker, D. W., & Eysenck, H. J. (1979). Nature and nurture. In H. J. Eysenck (Ed.), *The structure and measurement of intelligence* (pp. 102–174). Springer-Verlag.

Gilmore, J. H., Schmitt, J. E., Knickmeyer, R. C., Smith, J. K., Lin, W., Styner, M., Gerig, G., & Neale, M. C. (2010, Aug). Genetic and environmental contributions to neonatal brain structure: A twin study. *Hum Brain Mapp, 31*(8), 1174-1182. <https://doi.org/10.1002/hbm.20926>

Grotzinger, A. D., Briley, D. A., Engelhardt, L. E., Mann, F. D., Patterson, M. W., Tackett, J. L., Tucker-Drob, E. M., & Harden, K. P. (2018, Apr). Genetic and environmental influences on pubertal hormones in human hair across development. *Psychoneuroendocrinology, 90*, 76-84. <https://doi.org/10.1016/j.psyneuen.2018.02.005>

Grotzinger, A. D., Mann, F. D., Patterson, M. W., Herzhoff, K., Tackett, J. L., Tucker-Drob, E. M., & Paige Harden, K. (2018, Feb). Twin models of environmental and genetic influences on pubertal development, salivary testosterone, and estradiol in adolescence. *Clin Endocrinol (Oxf), 88*(2), 243-250. <https://doi.org/10.1111/cen.13522>

Gustavson, D. E., Hatton, S. N., Elman, J. A., Panizzon, M. S., Franz, C. E., Hagler, D. J., Jr., Fennema-Notestine, C., Eyler, L. T., McEvoy, L. K., Neale, M. C., Gillespie, N., Dale, A. M., Lyons, M. J., & Kremen, W. S. (2019, Jan 1). Predominantly global genetic influences on individual white matter tract microstructure. *Neuroimage, 184*, 871-880. <https://doi.org/10.1016/j.neuroimage.2018.10.016>

Haberstick, B. C., Schmitz, S., Young, S. E., & Hewitt, J. K. (2005, Jul). Contributions of genes and environments to stability and change in externalizing and internalizing problems during elementary and middle school. *Behav Genet, 35*(4), 381-396. <https://doi.org/10.1007/s10519-004-1747-5>

Haberstick, B. C., Schmitz, S., Young, S. E., & Hewitt, J. K. (2006, Nov). Genes and developmental stabiltiy of aggressive behavior problems at home and school in a community sample of twins aged 7-12. *Behav Genet, 36*(6), 809-819. <https://doi.org/10.1007/s10519-006-9092-5>

Harden, K. P., Kretsch, N., Tackett, J. L., & Tucker-Drob, E. M. (2014, Sep). Genetic and environmental influences on testosterone in adolescents: evidence for sex differences. *Dev Psychobiol, 56*(6), 1278-1289. <https://doi.org/10.1002/dev.21207>

Harris, J. A., Vernon, P. A., & Boomsma, D. I. (1998, May). The heritability of testosterone: a study of Dutch adolescent twins and their parents. *Behav Genet, 28*(3), 165-171. <https://doi.org/10.1023/a:1021466929053>

Haworth, C. M., Plomin, R., Carnell, S., & Wardle, J. (2008, Jul). Childhood obesity: genetic and environmental overlap with normal-range BMI. *Obesity (Silver Spring), 16*(7), 1585-1590. <https://doi.org/10.1038/oby.2008.240>

Haworth, C. M., Wright, M. J., Luciano, M., Martin, N. G., de Geus, E. J., van Beijsterveldt, C. E., Bartels, M., Posthuma, D., Boomsma, D. I., Davis, O. S., Kovas, Y., Corley, R. P., Defries, J. C., Hewitt, J. K., Olson, R. K., Rhea, S. A., Wadsworth, S. J., Iacono, W. G., McGue, M., Thompson, L. A., Hart, S. A., Petrill, S. A., Lubinski, D., & Plomin, R. (2010, Nov). The heritability of general cognitive ability increases linearly from childhood to young adulthood. *Mol Psychiatry, 15*(11), 1112-1120. <https://doi.org/10.1038/mp.2009.55>

Haworth, C. M., Wright, M. J., Martin, N. W., Martin, N. G., Boomsma, D. I., Bartels, M., Posthuma, D., Davis, O. S., Brant, A. M., Corley, R. P., Hewitt, J. K., Iacono, W. G., McGue, M., Thompson, L. A., Hart, S. A., Petrill, S. A., Lubinski, D., & Plomin, R. (2009, Jul). A twin study of the genetics of high cognitive ability selected from 11,000 twin pairs in six studies from four countries. *Behav Genet, 39*(4), 359-370. <https://doi.org/10.1007/s10519-009-9262-3>

Hewitt, J. K., Silberg, J. L., Neale, M. C., Eaves, L. J., & Erickson, M. (1992, May). The analysis of parental ratings of children's behavior using LISREL. *Behav Genet, 22*(3), 293-317. <http://www.ncbi.nlm.nih.gov/pubmed/1616461>

Hoekstra, R. A., Bartels, M., & Boomsma, D. I. (2006, Aug). Heritability of testosterone levels in 12-year-old twins and its relation to pubertal development. *Twin Res Hum Genet, 9*(4), 558-565. <https://doi.org/10.1375/183242706778025071>

Hur, Y. M., Kaprio, J., Iacono, W. G., Boomsma, D. I., McGue, M., Silventoinen, K., Martin, N. G., Luciano, M., Visscher, P. M., Rose, R. J., He, M., Ando, J., Ooki, S., Nonaka, K., Lin, C. C., Lajunen, H. R., Cornes, B. K., Bartels, M., van Beijsterveldt, C. E., Cherny, S. S., & Mitchell, K. (2008, Oct). Genetic influences on the difference in variability of height, weight and body mass index between Caucasian and East Asian adolescent twins. *Int J Obes (Lond), 32*(10), 1455-1467. <https://doi.org/10.1038/ijo.2008.144>

Iacono, W. G., Malone, S. M., & Vrieze, S. I. (2017, Jan). Endophenotype best practices. *Int J Psychophysiol, 111*, 115-144. <https://doi.org/10.1016/j.ijpsycho.2016.07.516>

Inderkum, A. P., & Tarokh, L. (2018, Mar 1). High heritability of adolescent sleep-wake behavior on free, but not school days: a long-term twin study. *Sleep, 41*(3). <https://doi.org/10.1093/sleep/zsy004>

Jansen, A. G., Mous, S. E., White, T., Posthuma, D., & Polderman, T. J. (2015, Mar). What twin studies tell us about the heritability of brain development, morphology, and function: a review. *Neuropsychol Rev, 25*(1), 27-46. <https://doi.org/10.1007/s11065-015-9278-9>

Jelenkovic, A., Sund, R., Hur, Y. M., Yokoyama, Y., Hjelmborg, J. V., Moller, S., Honda, C., Magnusson, P. K., Pedersen, N. L., Ooki, S., Aaltonen, S., Stazi, M. A., Fagnani, C., D'Ippolito, C., Freitas, D. L., Maia, J. A., Ji, F., Ning, F., Pang, Z., Rebato, E., Busjahn, A., Kandler, C., Saudino, K. J., Jang, K. L., Cozen, W., Hwang, A. E., Mack, T. M., Gao, W., Yu, C., Li, L., Corley, R. P., Huibregtse, B. M., Derom, C. A., Vlietinck, R. F., Loos, R. J., Heikkila, K., Wardle, J., Llewellyn, C. H., Fisher, A., McAdams, T. A., Eley, T. C., Gregory, A. M., He, M., Ding, X., Bjerregaard-Andersen, M., Beck-Nielsen, H., Sodemann, M., Tarnoki, A. D., Tarnoki, D. L., Knafo-Noam, A., Mankuta, D., Abramson, L., Burt, S. A., Klump, K. L., Silberg, J. L., Eaves, L. J., Maes, H. H., Krueger, R. F., McGue, M., Pahlen, S., Gatz, M., Butler, D. A., Bartels, M., van Beijsterveldt, T. C., Craig, J. M., Saffery, R., Dubois, L., Boivin, M., Brendgen, M., Dionne, G., Vitaro, F., Martin, N. G., Medland, S. E., Montgomery, G. W., Swan, G. E., Krasnow, R., Tynelius, P., Lichtenstein, P., Haworth, C. M., Plomin, R., Bayasgalan, G., Narandalai, D., Harden, K. P., Tucker-Drob, E. M., Spector, T., Mangino, M., Lachance, G., Baker, L. A., Tuvblad, C., Duncan, G. E., Buchwald, D., Willemsen, G., Skytthe, A., Kyvik, K. O., Christensen, K., Oncel, S. Y., Aliev, F., Rasmussen, F., Goldberg, J. H., Sorensen, T. I., Boomsma, D. I., Kaprio, J., & Silventoinen, K. (2016, Jun 23). Genetic and environmental influences on height from infancy to early adulthood: An individual-based pooled analysis of 45 twin cohorts. *Sci Rep, 6*, 28496. <https://doi.org/10.1038/srep28496>

Jha, S. C., Xia, K., Schmitt, J. E., Ahn, M., Girault, J. B., Murphy, V. A., Li, G., Wang, L., Shen, D., Zou, F., Zhu, H., Styner, M., Knickmeyer, R. C., & Gilmore, J. H. (2018, Dec). Genetic influences on neonatal cortical thickness and surface area. *Hum Brain Mapp, 39*(12), 4998-5013. <https://doi.org/10.1002/hbm.24340>

Jutten, K., Mainz, V., Gauggel, S., Patel, H. J., Binkofski, F., Wiesmann, M., Clusmann, H., & Na, C. H. (2019). Diffusion Tensor Imaging Reveals Microstructural Heterogeneity of Normal-Appearing White Matter and Related Cognitive Dysfunction in Glioma Patients. *Front Oncol, 9*, 536. <https://doi.org/10.3389/fonc.2019.00536>

Kendler, K. S., & Neale, M. C. (2010, Aug). Endophenotype: a conceptual analysis. *Mol Psychiatry, 15*(8), 789-797. <https://doi.org/10.1038/mp.2010.8>

King, M. J., Katz, D. P., Thompson, L. A., & Macnamara, B. N. (2019). Genetic and environmental influences on spatial reasoning: A meta-analysis of twin studies. *Intelligence, 73*, 65–77.

Kochunov, P., Jahanshad, N., Marcus, D., Winkler, A., Sprooten, E., Nichols, T. E., Wright, S. N., Hong, L. E., Patel, B., Behrens, T., Jbabdi, S., Andersson, J., Lenglet, C., Yacoub, E., Moeller, S., Auerbach, E., Ugurbil, K., Sotiropoulos, S. N., Brouwer, R. M., Landman, B., Lemaitre, H., den Braber, A., Zwiers, M. P., Ritchie, S., van Hulzen, K., Almasy, L., Curran, J., deZubicaray, G. I., Duggirala, R., Fox, P., Martin, N. G., McMahon, K. L., Mitchell, B., Olvera, R. L., Peterson, C., Starr, J., Sussmann, J., Wardlaw, J., Wright, M., Boomsma, D. I., Kahn, R., de Geus, E. J., Williamson, D. E., Hariri, A., van 't Ent, D., Bastin, M. E., McIntosh, A., Deary, I. J., Hulshoff Pol, H. E., Blangero, J., Thompson, P. M., Glahn, D. C., & Van Essen, D. C. (2015, May 1). Heritability of fractional anisotropy in human white matter: a comparison of Human Connectome Project and ENIGMA-DTI data. *Neuroimage, 111*, 300-311. <https://doi.org/10.1016/j.neuroimage.2015.02.050>

Koenis, M. M., Brouwer, R. M., van Baal, G. C., van Soelen, I. L., Peper, J. S., van Leeuwen, M., Delemarre-van de Waal, H. A., Boomsma, D. I., & Hulshoff Pol, H. E. (2013, Mar). Longitudinal study of hormonal and physical development in young twins. *J Clin Endocrinol Metab, 98*(3), E518-527. <https://doi.org/10.1210/jc.2012-3361>

Lee, S. J., Zhang, J., Neale, M. C., Styner, M., Zhu, H., & Gilmore, J. H. (2019, Mar). Quantitative tract-based white matter heritability in 1- and 2-year-old twins. *Hum Brain Mapp, 40*(4), 1164-1173. <https://doi.org/10.1002/hbm.24436>

Lenroot, R. K., Schmitt, J. E., Ordaz, S. J., Wallace, G. L., Neale, M. C., Lerch, J. P., Kendler, K. S., Evans, A. C., & Giedd, J. N. (2009, Jan). Differences in genetic and environmental influences on the human cerebral cortex associated with development during childhood and adolescence. *Hum Brain Mapp, 30*(1), 163-174. <https://doi.org/10.1002/hbm.20494>

Li, H., Ji, C., Yang, L., & Zhuang, C. (2017, Jun). Heritability of serum dehydroepiandrosterone sulphate levels and pubertal development in 6 approximately 18-year-old girls: a twin study. *Ann Hum Biol, 44*(4), 325-331. <https://doi.org/10.1080/03014460.2016.1240232>

Liu, Q., Yu, C., Gao, W., Cao, W., Lyu, J., Wang, S., Pang, Z., Cong, L., Dong, Z., Wu, F., Wang, H., Wu, X., Jiang, G., Wang, B., & Li, L. (2015, Oct). Genetic and Environmental Effects on Weight, Height, and BMI Under 18 Years in a Chinese Population-Based Twin Sample. *Twin Res Hum Genet, 18*(5), 571-580. <https://doi.org/10.1017/thg.2015.63>

Loehlin, J. C., & Nichols, R. C. (1976). *Heredity, environment, and personality: A study of 850 sets of twins*. University of Texas Press.

Maes, H. H., Neale, M. C., & Eaves, L. J. (1997, Jul). Genetic and environmental factors in relative body weight and human adiposity. *Behav Genet, 27*(4), 325-351. <http://www.ncbi.nlm.nih.gov/pubmed/9519560>

Malone, S. M., & Iacono, W. G. (2002, Sep). Error rate on the antisaccade task: heritability and developmental change in performance among preadolescent and late-adolescent female twin youth. *Psychophysiology, 39*(5), 664-673. <https://www.ncbi.nlm.nih.gov/pubmed/12236334>

McGue, M., Bouchard, T. J. J., Iacono, W. G., & Lykken, D. T. (1993). Behavioral genetics of cognitive ability: A life-span perspective. In R. Plomin & G. E. McClearn (Eds.), *Nature, Nurture, and Psychology* (pp. 59–76). American Psychological Association Press.

McKay, D. R., Winkler, A. M., Kochunov, P., Knowles, E. E. M., Sprooten, E., & Fox, P. T. (2015). Genetic Influence on the Human Brain. In R. Duggirala, L. Almasy, S. Williams-Blangero, S. F. D. Paul, & C. Kole (Eds.), *Genome Mapping and Genomics in Human and Non-Human Primates.* (pp. 247–258). Springer Berlin Heidelberg.

Miyake, A., Friedman, N. P., Emerson, M. J., Witzki, A. H., Howerter, A., & Wager, T. D. (2000, Aug). The unity and diversity of executive functions and their contributions to complex "Frontal Lobe" tasks: a latent variable analysis. *Cogn Psychol, 41*(1), 49-100. <https://doi.org/10.1006/cogp.1999.0734>

Mori, S., & Zhang, J. (2006, Sep 7). Principles of diffusion tensor imaging and its applications to basic neuroscience research. *Neuron, 51*(5), 527-539. <https://doi.org/10.1016/j.neuron.2006.08.012>

Nan, C., Guo, B., Warner, C., Fowler, T., Barrett, T., Boomsma, D., Nelson, T., Whitfield, K., Beunen, G., Thomis, M., Maes, H. H., Derom, C., Ordonana, J., Deeks, J., & Zeegers, M. (2012, Apr). Heritability of body mass index in pre-adolescence, young adulthood and late adulthood. *Eur J Epidemiol, 27*(4), 247-253. <https://doi.org/10.1007/s10654-012-9678-6>

Neale, M. C., & Stevenson, J. (1989, Mar). Rater bias in the EASI temperament scales: a twin study. *J Pers Soc Psychol, 56*(3), 446-455. <https://doi.org/10.1037//0022-3514.56.3.446>

Neisser, U., Boodoo, G., Bouchard, T. J. J., Boykin, A., Brody, N., Ceci, S. J., Halpern, D. E., Loehlin, J. C., Perloff, R., Sternberg, R. J., & Urbina, S. (1996). Intelligence: Knowns and unknowns. *American Psychologist, 51*, 77–101.

Panizzon, M. S., Fennema-Notestine, C., Eyler, L. T., Jernigan, T. L., Prom-Wormley, E., Neale, M., Jacobson, K., Lyons, M. J., Grant, M. D., Franz, C. E., Xian, H., Tsuang, M., Fischl, B., Seidman, L., Dale, A., & Kremen, W. S. (2009, Nov). Distinct genetic influences on cortical surface area and cortical thickness. *Cereb Cortex, 19*(11), 2728-2735. <https://doi.org/10.1093/cercor/bhp026>

Peper, J. S., Brouwer, R. M., Boomsma, D. I., Kahn, R. S., & Hulshoff Pol, H. E. (2007, Jun). Genetic influences on human brain structure: a review of brain imaging studies in twins. *Hum Brain Mapp, 28*(6), 464-473. <https://doi.org/10.1002/hbm.20398>

Peper, J. S., Schnack, H. G., Brouwer, R. M., Van Baal, G. C., Pjetri, E., Szekely, E., van Leeuwen, M., van den Berg, S. M., Collins, D. L., Evans, A. C., Boomsma, D. I., Kahn, R. S., & Hulshoff Pol, H. E. (2009, Jul). Heritability of regional and global brain structure at the onset of puberty: a magnetic resonance imaging study in 9-year-old twin pairs. *Hum Brain Mapp, 30*(7), 2184-2196. <https://doi.org/10.1002/hbm.20660>

Perez, A., Gabriel, K., Nehme, E. K., Mandell, D. J., & Hoelscher, D. M. (2015, Jul 27). Measuring the bias, precision, accuracy, and validity of self-reported height and weight in assessing overweight and obesity status among adolescents using a surveillance system. *Int J Behav Nutr Phys Act, 12 Suppl 1*, S2. <https://doi.org/10.1186/1479-5868-12-S1-S2>

Pinto, C. B., Bielefeld, J., Jabakhanji, R., Reckziegel, D., Griffith, J. W., & Apkarian, A. V. (2020). Neural and Genetic Bases for Human Ability Traits. *Front Hum Neurosci, 14*, 609170. <https://doi.org/10.3389/fnhum.2020.609170>

Plomin, R., & Deary, I. J. (2015, Feb). Genetics and intelligence differences: five special findings. *Mol Psychiatry, 20*(1), 98-108. <https://doi.org/10.1038/mp.2014.105>

Plomin, R., & DeFries, J. C. (1980). Genetics and intelligence: Recent data. Intelligence. *4*, 15–24.

Polderman, T. J., Benyamin, B., de Leeuw, C. A., Sullivan, P. F., van Bochoven, A., Visscher, P. M., & Posthuma, D. (2015, Jul). Meta-analysis of the heritability of human traits based on fifty years of twin studies. *Nat Genet, 47*(7), 702-709. <https://doi.org/10.1038/ng.3285>

Posthuma, D., De Geus, E. J., Baare, W. F., Hulshoff Pol, H. E., Kahn, R. S., & Boomsma, D. I. (2002, Feb). The association between brain volume and intelligence is of genetic origin. *Nat Neurosci, 5*(2), 83-84. <https://doi.org/10.1038/nn0202-83>

Rusterholz, T., Hamann, C., Markovic, A., Schmidt, S. J., Achermann, P., & Tarokh, L. (2018, Oct 24). Nature and Nurture: Brain Region-Specific Inheritance of Sleep Neurophysiology in Adolescence. *J Neurosci, 38*(43), 9275-9285. <https://doi.org/10.1523/JNEUROSCI.0945-18.2018>

Schmitt, J. E., Neale, M. C., Clasen, L. S., Liu, S., Seidlitz, J., Pritikin, J. N., Chu, A., Wallace, G. L., Lee, N. R., Giedd, J. N., & Raznahan, A. (2019, Apr 17). A Comprehensive Quantitative Genetic Analysis of Cerebral Surface Area in Youth. *J Neurosci, 39*(16), 3028-3040. <https://doi.org/10.1523/JNEUROSCI.2248-18.2019>

Schmitt, J. E., Neale, M. C., Fassassi, B., Perez, J., Lenroot, R. K., Wells, E. M., & Giedd, J. N. (2014, May 6). The dynamic role of genetics on cortical patterning during childhood and adolescence. *Proc Natl Acad Sci U S A, 111*(18), 6774-6779. <https://doi.org/10.1073/pnas.1311630111>

Schmitt, J. E., Raznahan, A., Clasen, L. S., Wallace, G. L., Pritikin, J. N., Lee, N. R., Giedd, J. N., & Neale, M. C. (2019, Dec 17). The Dynamic Associations Between Cortical Thickness and General Intelligence are Genetically Mediated. *Cereb Cortex, 29*(11), 4743-4752. <https://doi.org/10.1093/cercor/bhz007>

Silventoinen, K., Bartels, M., Posthuma, D., Estourgie-van Burk, G. F., Willemsen, G., van Beijsterveldt, T. C., & Boomsma, D. I. (2007, Apr). Genetic regulation of growth in height and weight from 3 to 12 years of age: a longitudinal study of Dutch twin children. *Twin Res Hum Genet, 10*(2), 354-363. <https://doi.org/10.1375/twin.10.2.354>

Silventoinen, K., Jelenkovic, A., Sund, R., Hur, Y. M., Yokoyama, Y., Honda, C., Hjelmborg, J., Moller, S., Ooki, S., Aaltonen, S., Ji, F., Ning, F., Pang, Z., Rebato, E., Busjahn, A., Kandler, C., Saudino, K. J., Jang, K. L., Cozen, W., Hwang, A. E., Mack, T. M., Gao, W., Yu, C., Li, L., Corley, R. P., Huibregtse, B. M., Christensen, K., Skytthe, A., Kyvik, K. O., Derom, C. A., Vlietinck, R. F., Loos, R. J., Heikkila, K., Wardle, J., Llewellyn, C. H., Fisher, A., McAdams, T. A., Eley, T. C., Gregory, A. M., He, M., Ding, X., Bjerregaard-Andersen, M., Beck-Nielsen, H., Sodemann, M., Tarnoki, A. D., Tarnoki, D. L., Stazi, M. A., Fagnani, C., D'Ippolito, C., Knafo-Noam, A., Mankuta, D., Abramson, L., Burt, S. A., Klump, K. L., Silberg, J. L., Eaves, L. J., Maes, H. H., Krueger, R. F., McGue, M., Pahlen, S., Gatz, M., Butler, D. A., Bartels, M., van Beijsterveldt, T. C., Craig, J. M., Saffery, R., Freitas, D. L., Maia, J. A., Dubois, L., Boivin, M., Brendgen, M., Dionne, G., Vitaro, F., Martin, N. G., Medland, S. E., Montgomery, G. W., Chong, Y., Swan, G. E., Krasnow, R., Magnusson, P. K., Pedersen, N. L., Tynelius, P., Lichtenstein, P., Haworth, C. M., Plomin, R., Bayasgalan, G., Narandalai, D., Harden, K. P., Tucker-Drob, E. M., Oncel, S. Y., Aliev, F., Spector, T., Mangino, M., Lachance, G., Baker, L. A., Tuvblad, C., Duncan, G. E., Buchwald, D., Willemsen, G., Rasmussen, F., Goldberg, J. H., Sorensen, T., Boomsma, D. I., & Kaprio, J. (2016, Aug). Genetic and environmental effects on body mass index from infancy to the onset of adulthood: an individual-based pooled analysis of 45 twin cohorts participating in the COllaborative project of Development of Anthropometrical measures in Twins (CODATwins) study. *Am J Clin Nutr, 104*(2), 371-379. <https://doi.org/10.3945/ajcn.116.130252>

Silventoinen, K., Jelenkovic, A., Sund, R., Yokoyama, Y., Hur, Y. M., Cozen, W., Hwang, A. E., Mack, T. M., Honda, C., Inui, F., Iwatani, Y., Watanabe, M., Tomizawa, R., Pietilainen, K. H., Rissanen, A., Siribaddana, S. H., Hotopf, M., Sumathipala, A., Rijsdijk, F., Tan, Q., Zhang, D., Pang, Z., Piirtola, M., Aaltonen, S., Oncel, S. Y., Aliev, F., Rebato, E., Hjelmborg, J. B., Christensen, K., Skytthe, A., Kyvik, K. O., Silberg, J. L., Eaves, L. J., Cutler, T. L., Ordonana, J. R., Sanchez-Romera, J. F., Colodro-Conde, L., Song, Y. M., Yang, S., Lee, K., Franz, C. E., Kremen, W. S., Lyons, M. J., Busjahn, A., Nelson, T. L., Whitfield, K. E., Kandler, C., Jang, K. L., Gatz, M., Butler, D. A., Stazi, M. A., Fagnani, C., D'Ippolito, C., Duncan, G. E., Buchwald, D., Martin, N. G., Medland, S. E., Montgomery, G. W., Jeong, H. U., Swan, G. E., Krasnow, R., Magnusson, P. K., Pedersen, N. L., Dahl Aslan, A. K., McAdams, T. A., Eley, T. C., Gregory, A. M., Tynelius, P., Baker, L. A., Tuvblad, C., Bayasgalan, G., Narandalai, D., Spector, T. D., Mangino, M., Lachance, G., Burt, S. A., Klump, K. L., Harris, J. R., Brandt, I., Nilsen, T. S., Krueger, R. F., McGue, M., Pahlen, S., Corley, R. P., Huibregtse, B. M., Bartels, M., van Beijsterveldt, C. E., Willemsen, G., Goldberg, J. H., Rasmussen, F., Tarnoki, A. D., Tarnoki, D. L., Derom, C. A., Vlietinck, R. F., Loos, R. J., Hopper, J. L., Sung, J., Maes, H. H., Turkheimer, E., Boomsma, D. I., Sorensen, T. I., & Kaprio, J. (2017, Aug). Differences in genetic and environmental variation in adult BMI by sex, age, time period, and region: an individual-based pooled analysis of 40 twin cohorts. *Am J Clin Nutr, 106*(2), 457-466. <https://doi.org/10.3945/ajcn.117.153643>

Sletten, T. L., Rajaratnam, S. M., Wright, M. J., Zhu, G., Naismith, S., Martin, N. G., & Hickie, I. (2013, Nov 1). Genetic and environmental contributions to sleep-wake behavior in 12-year-old twins. *Sleep, 36*(11), 1715-1722. <https://doi.org/10.5665/sleep.3136>

Soares, J. M., Marques, P., Alves, V., & Sousa, N. (2013). A hitchhiker's guide to diffusion tensor imaging. *Front Neurosci, 7*, 31. <https://doi.org/10.3389/fnins.2013.00031>

Stins, J. F., van Baal, G. C., Polderman, T. J., Verhulst, F. C., & Boomsma, D. I. (2004, Dec 3). Heritability of Stroop and flanker performance in 12-year old children. *BMC Neurosci, 5*, 49. <https://doi.org/10.1186/1471-2202-5-49>

Strike, L. T., Hansell, N. K., Couvy-Duchesne, B., Thompson, P. M., de Zubicaray, G. I., McMahon, K. L., & Wright, M. J. (2019, Mar 1). Genetic Complexity of Cortical Structure: Differences in Genetic and Environmental Factors Influencing Cortical Surface Area and Thickness. *Cereb Cortex, 29*(3), 952-962. <https://doi.org/10.1093/cercor/bhy002>

Swagerman, S. C., Brouwer, R. M., de Geus, E. J., Hulshoff Pol, H. E., & Boomsma, D. I. (2014, Nov). Development and heritability of subcortical brain volumes at ages 9 and 12. *Genes Brain Behav, 13*(8), 733-742. <https://doi.org/10.1111/gbb.12182>

Teeuw, J., Brouwer, R. M., Koenis, M. M. G., Swagerman, S. C., Boomsma, D. I., & Hulshoff Pol, H. E. (2019, Mar 1). Genetic Influences on the Development of Cerebral Cortical Thickness During Childhood and Adolescence in a Dutch Longitudinal Twin Sample: The Brainscale Study. *Cereb Cortex, 29*(3), 978-993. <https://doi.org/10.1093/cercor/bhy005>

Thompson, P. M., Cannon, T. D., Narr, K. L., van Erp, T., Poutanen, V. P., Huttunen, M., Lonnqvist, J., Standertskjold-Nordenstam, C. G., Kaprio, J., Khaledy, M., Dail, R., Zoumalan, C. I., & Toga, A. W. (2001, Dec). Genetic influences on brain structure. *Nat Neurosci, 4*(12), 1253-1258. <https://doi.org/10.1038/nn758>

Thompson, W. K., Barch, D. M., Bjork, J. M., Gonzalez, R., Nagel, B. J., Nixon, S. J., & Luciana, M. (2019, Apr). The structure of cognition in 9 and 10 year-old children and associations with problem behaviors: Findings from the ABCD study's baseline neurocognitive battery. *Dev Cogn Neurosci, 36*, 100606. <https://doi.org/10.1016/j.dcn.2018.12.004>

Turkheimer, E., & Horn, E. E. (2014). Interactions between socioeconomic status and components of variation in cognitive ability. In D. Finkel & C. A. Reynolds (Eds.), *Behavior genetics of cognition across the lifespan* (pp. 41–68). Springer.

van der Valk, J. C., van den Oord, E. J., Verhulst, F. C., & Boomsma, D. I. (2003, Jul). Using shared and unique parental views to study the etiology of 7-year-old twins' internalizing and externalizing problems. *Behav Genet, 33*(4), 409-420. <https://doi.org/10.1023/a:1025369525924>

Van Hulle, C. A., Moore, M. N., Shirtcliff, E. A., Lemery-Chalfant, K., & Goldsmith, H. H. (2015, May). Genetic and Environmental Contributions to Covariation Between DHEA and Testosterone in Adolescent Twins. *Behav Genet, 45*(3), 324-340. <https://doi.org/10.1007/s10519-015-9709-7>

van Soelen, I. L., Brouwer, R. M., van Leeuwen, M., Kahn, R. S., Hulshoff Pol, H. E., & Boomsma, D. I. (2011, Apr). Heritability of verbal and performance intelligence in a pediatric longitudinal sample. *Twin Res Hum Genet, 14*(2), 119-128. <https://doi.org/10.1375/twin.14.2.119>

Wadsworth, S. J., Corley, R. P., & DeFries, J. C. (2014). Cognitive abilities in childhood and adolescence. In D. Finkel & C. A. Reynolds (Eds.), *Behavior genetics of cognition across the lifespan* (pp. 3-40). Springer.

Wallace, G. L., Eric Schmitt, J., Lenroot, R., Viding, E., Ordaz, S., Rosenthal, M. A., Molloy, E. A., Clasen, L. S., Kendler, K. S., Neale, M. C., & Giedd, J. N. (2006, Oct). A pediatric twin study of brain morphometry. *J Child Psychol Psychiatry, 47*(10), 987-993. <https://doi.org/10.1111/j.1469-7610.2006.01676.x>

Yoon, U., Perusse, D., & Evans, A. C. (2012, Sep 18). Mapping genetic and environmental influences on cortical surface area of pediatric twins. *Neuroscience, 220*, 169-178. <https://doi.org/10.1016/j.neuroscience.2012.06.030>

Zhang, Z., Descoteaux, M., Zhang, J., Girard, G., Chamberland, M., Dunson, D., Srivastava, A., & Zhu, H. (2018, May 15). Mapping population-based structural connectomes. *Neuroimage, 172*, 130-145. <https://doi.org/10.1016/j.neuroimage.2017.12.064>
